# Adverse changes in close social ties reduce fruit and vegetable intake in aging adults: a prospective gender-sensitive study of the Canadian longitudinal study on aging (CLSA)

**DOI:** 10.1186/s12966-025-01807-7

**Published:** 2025-08-13

**Authors:** Sanaz Mehranfar, Gilciane Ceolin, Rana Madani Civi, Heather Keller, Rachel A. Murphy, Tamara R. Cohen, Annalijn I. Conklin

**Affiliations:** 1 Faculty of Land and Food Systems, The University of British Columbia, Vancouver, BC Canada; 2 Faculty of Pharmaceutical Sciences, The University of British Columbia, Vancouver, BC Canada; 3 Department of Kinesiology and Health Sciences, University of Waterloo, Waterloo, ON Canada; 4 Schlegel-UW Research Institute for Aging, Waterloo, ON Canada; 5 Cancer Control Research, BC Cancer Research Institute, Vancouver, BC Canada; 6 School of Population and Public Health, Faculty of Medicine, The University of British Columbia, Vancouver, BC Canada; 7 BC Children’s Hospital Research Institute, BC Children’s Hospital, Vancouver, BC Canada; 8 Centre for Advancing Health Outcomes, Providence Health Care Research Institute, St. Paul’s Hospital, Vancouver, BC Canada; 9 Edwin S.H. Leong Centre for Healthy Aging, Faculty of Medicine, The University of British Columbia, Vancouver, BC Canada; 10 Faculty of Pharmaceutical Sciences, The University of British Columbia, Room 4623, 2405 Wesbrook Mall, Vancouver, V6T 1Z3 Canada

**Keywords:** Social relationships, Fruits and vegetables, Gender, Longitudinal, CLSA

## Abstract

**Background:**

Close social ties are known to increase survival, reduce chronic diseases, and promote healthful eating. Little research has explored whether adverse changes in these relationships lead to less healthful eating in older adults, with attention to gender differences.

**Methods:**

Prospective study using 3 waves of the Canadian Longitudinal Study on Aging (CLSA) in a sample of middle-age and older adults (45–85 y) reporting daily intake of fruit or vegetable (F/V) intake (at least one time per day) at baseline using dietary data collected by CLSA’s Short Diet Questionnaire. We used multivariable multilevel logistic regression with interaction terms (social tie x gender) to determine whether adverse changes in close social ties (marital status and living arrangement) between baseline (2011–2015) and follow-up 1 (2015–2018) led to developing less healthful eating measured by non-daily intake of F/V at follow-up 2 (2018–2021) (*n* = 15,672); models adjusted for biological, behavioural, socioeconomic, and socio-political confounders.

**Results:**

Distinct transitions by gender precipitated a change from daily F/V intake (healthful eating) to less frequent intakes (unhealthful eating). Compared to women remaining partnered, women remaining non-partnered over 3 years had 21% higher odds of reducing healthful intake of vegetables at 6-year follow-up (OR 1.21 [95% CI: 1.07, 1.38]). Becoming divorced increased the odds of reducing healthful intake of fruits among women (1.76 [1.16, 2.66]) compared to referent. Women remaining lone-living were less likely to reduce healthful fruit intake (0.86 [0.74, 0.99]), compared to remaining co-living. Compared to men remaining partnered, men who became divorced or widowed had 91% greater odds of reducing healthful vegetable intake (1.91 [1.25, 2.92] and 1.91 [1.17, 3.13], respectively). Men who remained non-partnered or became widowed were also more likely to reduce healthful fruit intake (1.20 [1.03, 1.41] and 1.99 [1.26, 3.15], respectively), compared to referent. Finally, men who became lone-living and co-living were more likely to reduce healthful intakes of vegetables (1.42 [1.06, 1.91] and 1.55 [1.04, 2.32]) and fruits (1.48 [1.11, 1.96] and 1.48 [1.00, 2.18]), compared to men remaining co-living.

**Conclusions:**

Findings showed that adverse changes in close social ties led to the development of less healthful eating among aging adults in Canada, and these prospective associations appeared to be gendered. Public health and nutrition interventions should consider the social context as a risk factor to address gender disparities in food intake in the aging population.

**Supplementary Information:**

The online version contains supplementary material available at 10.1186/s12966-025-01807-7.

## Background

Globally, demographic profiles are shifting towards an older population [1]; in particular, the proportion of Canada’s population aged 65 and older is expected to grow from 18.5% in 2021 to 23.1% by 2043 and reach 25.9% by 2068 [2]. Social connections are known to improve survival [3] and health outcomes [4] including reducing cardiovascular events [5, 6], but these change as people age. Older adults (aged 65+) are most vulnerable to social isolation from changes to marital status and/or living arrangement [7]. Unlike married individuals, older people who experience adverse life-course transitions like becoming divorced or widowed have greater risks of poor mental health [8], mortality [9, 10], cardiovascular diseases (CVD) [11] and cancer [12]. Lone-living may also impact health and wellbeing, as cohabitation can provide tangible support for maintaining health [13–15]. One mechanism that is postulated to underpin the impact of social ties on physical health is health-related behaviours including diet [16, 17].

Consuming a high-quality diet, particularly one rich in fruits and vegetables (FV), is associated with living longer [18], and also with a lower risk of chronic diseases, cognitive decline, and frailty among older adults [19]. Importantly, social factors are a prominent influence on the diets of older adults [20–23]. Indeed, different types of social ties are associated with healthful eating among older adults [24, 25]. Marriage may be the strongest social influence on food choices and healthful eating habits, as older adults who are married have greater levels of diet quality compared to their non-married (particularly widowed) counterparts [26]. Cross-sectional studies also show a link between living arrangement (co-living versus lone-living) and diet quality in older adults [24, 27, 28]. Although some longitudinal studies of change in social ties and change in FV intake among older adults exist, this small literature focuses entirely on marital transitions [29]. Overall, marital dissolution seems to reduce intakes with greater reductions seen among older men [30–36]. Existing longitudinal evidence, however, is limited because it assesses marital transitions over the same timeframe as changes in diet quality markers and therefore lacks temporality to support causal inference.

While diets appear most concordant among close social ties [37], there are important gender differences. Wives contribute more to their husbands’ diet quality than husbands contribute to theirs [38], and women often prioritize their family members’ dietary preferences over their own [39]. One longitudinal study showed that becoming widowed decreases FV intakes among men, whereas becoming divorced/separated decreases vegetable intake among women [32]. Another longitudinal study found marital dissolution adversely affects vegetable consumption more among men than women [36]. Two cross-sectional studies on living alone also indicate gender differences in the associations with poor dietary quality [40] or unhealthful eating, such as meal skipping or eating out [41]. Since women and men differ in the size and type of social connections they maintain, and how changes in these connections may affect health-related outcomes [27, 42], it is imperative to apply a sex- and gender-based perspective when examining the broader determinants of healthful eating [43]. This population-based study aimed to prospectively examine gender-specific changes in two close social ties (living arrangement and marital status) in relation to changes in healthful eating among older adults, leveraging multiple waves of a Canadian aging cohort. We hypothesized that adverse changes in marital status or living arrangements (losses or persistent lack) would reduce daily intake of fruits or vegetables (F/V) in a gender-specific manner.

## Methods

### Study population

We used three waves of data—baseline (BL, 2011–2015), follow-up 1 (FU1, 2015–2018), and follow-up 2 (FU2, 2018–2021)—from 30,097 predominantly White, non-institutionalized, middle- and older-aged participants (45 years and above) in the Canadian Longitudinal Study on Aging (CLSA) Comprehensive Cohort, a stratified random sample of community-dwelling individuals [44]. Details of the CLSA cohort are given in the Supplemental Methods. We excluded CLSA participants who did not consume fruits (26%) or vegetables (36%) at least one time per day at baseline as we were interested in the development of less healthful eating. Thus, our eligible sample was restricted to people reporting intakes of one time or more per day of fruit (*n* = 21,178, 74%) or vegetables (*n* = 18,333, 64%) at BL and who had complete information for all included covariables and either exposure. For those with daily fruit intake at BL, the eligible sample for marital status was *n* = 18,081 (10,192 women; 7889 men) and for living arrangement was 18,019 (10,155 women; 7864 men); for those with daily vegetable intake, eligible samples were 15,727 (9206 women; 6521 men) and 15,672 (9175 women; 6497 men), respectively. Details of sample selection process are given in Supplemental Fig. S1.

### Changes in fruit or vegetable intake

Our primary outcome was adverse change from healthful eating at baseline (2011–2015) to less healthful eating at 6-year follow-up (2018–2021). Healthful eating was defined as the intake of fruits or vegetables (F/V) at least once per day, reflecting a frequency rather than serving size in line with Canada’s *Food Guide* [45]; healthful eating was operationalized using the recommended daily consumption of fruits and/or vegetables since it is an established indicator of diet quality and known to reduce the risk of morbidity and mortality [46, 47]. To assess fruit intake less than once per day (non-daily fruit) and vegetable intake less than once per day (non-daily vegetable), we used dietary data collected by CLSA’s Short Diet Questionnaire (SDQ) which is a 36-item validated non-quantitative food frequency questionnaire (FFQ) focused on foods and nutrients of concern in older populations [48]. CLSA responses for consumption of food items were daily, weekly, monthly or yearly, followed by another question of “how many times” for that frequency response (e.g., twice a day, three times a week, once a month); the SDQ had five questions on fruit, green vegetables, potatoes, carrots, and other vegetables that were used in this analysis. CLSA converted all SDQ responses into times per day. These were summarized into daily and non-daily intake of either vegetables (based on 4 questions) or fruits (based on 1 question). Details on the SDQ items, the process of dichotomization and the handling outliers are given in the Supplemental Methods; Supplemental Table S1 gives our coding method for mock CLSA responses.

### Transitions in marital status and living arrangement

To assess changes in two close social ties, we used CLSA data from BL and FU1 on self-reported marital status and living arrangement for the construction of ‘transition’ variables. We combined 6 responses for marital status into 4 categories at both time points (partnered (married/living as married), single, widowed, and divorced/separated) to then derive a marital transition variable with 6 categories in line with previous research [49, 50]. Marital transitions were: *remained partnered* (partnered/married at BL and FU1, reference); *remained non-partnered* (divorced/separated, widowed or single at BL and FU1); *became divorced/separated* (partnered/married at BL, divorced/separated at FU1); *became widowed* (partnered/married at BL, widowed at FU1); *became partnered* (widowed, divorced/separated or single at BL, partnered at FU1); *uncertain transitions* (e.g., single to divorced, widowed to single, etc., see details in Supplemental Table S2). Community-dwelling participants in the CLSA were asked ‘how many people, not including yourself, currently live in your household?’ (this was followed by details about their relationship to the participant, e.g., spouse, parent, child, grandparent, grandchild, etc.). We used CLSA responses to assess living arrangement by categorizing participants as living alone (lone-living) or living with someone else (co-living) at BL and FU1, and then constructed a transition variable for living arrangement: *remained co-living* (reference), *remained lone-living*, *became lone-living*, and *became co-living*. Cohabitation changes were moderately associated with marital transitions (Cramer’s V = 0.56). As change in each indicator of close social ties encompasses gain, loss, and no change, we included stable transitions in both derived variables.

### Covariables

Study duration was calculated in months between BL and FU2. Supplemental Fig. S2 shows the Directed Acyclic Graph (DAG) we created with DAGitty software (version 3.0; Nijmegen, GE, The Netherlands) [51], to identify theoretical, plausible, and known confounders [30–36, 52, 53] which included study duration, baseline age, body mass index (BMI), ≥ 1 chronic conditions, education, household income, home-ownership, rural/urban location, alcohol, smoking, and sleep (details in Supplemental Methods). Given the nested nature of CLSA data, we also included socio-political confounders from Statistics Canada for provincial gross domestic product (GDP) [54], provincial expenditure on social protection [55], and provincial food insecurity [56].

### Sex & gender considerations

CLSA asked participants at BL, ‘are you male or female?’, which represents biological sex or gender identity or both. At FU1, CLSA also asked about participants’ “gender identity” (male, female, transgender man/transman, transgender woman/transwoman, genderqueer, and other), with very few non-cisgender observations (*n* = 40); gender identity was very strongly correlated with male/female (Cramer’s V = 0.998). We interpret results through a lens of gender (i.e. gender identity, gender roles, gender relations, and institutionalized gender) [57].

### Statistical analysis

Means (SD) and frequencies (%) were used to describe sample characteristics across transition categories of two close social ties. Descriptive analysis used CLSA survey inflation weights to produce estimates that represent the entire population of Canadians aged 45 to 85 residing in the geographic area of the data collection sites [58]. The main analysis examined the gender-specific prospective associations between changes in two close social ties and development of non-daily intake of F/V at FU2 in the sample of CLSA participants reporting F/V intake of once per day or more at BL. A series of multivariable-adjusted mixed-effect logistic regressions used a random coefficient model to account for the natural nesting of CLSA data (individuals nested within geographic strata) and included an interaction term (social tie x gender) to generate ORs specific to women and men. Model A included study duration, baseline age, BMI, and chronic condition; Model B further adjusted for socioeconomic status indicators (education, household income, home-ownership, and rural/urban location); Model C further adjusted for behavioural factors (alcohol, smoking and sleep deprivation); and Model D further adjusted for province-level covariables (provincial GDP, provincial expenditure on social protection, and provincial food insecurity). We assessed multicollinearity using variance inflation factor (VIF) and found no collinearity except for provincial GDP (VIF = 11.69) which was retained in the model based on our DAG.

We ran duplicate regressions using reverse coding of the interaction term (women = 0 and men = 1; women = 1 and men = 0) to provide estimates for each reference group from the same model. Inferential statistics did not include analytic weights because CLSA does not provide sampling weights at both the primary sampling unit (individual) and the provincial level (geographic strata) [59]. Model fit was tested using likelihood ratio tests and Akaike and Bayesian Information Criteria. Post-estimation of regression coefficients then calculated the predicted probability of non-daily intake of F/V associated with each transition category in both close social ties (STATA ‘*margins*’ command); average predicted probabilities were plotted for ease of interpretation (‘*marginsplot*’). Final results were reported as Odds Ratios (OR) and average predicted probability, with 95% confidence intervals (95% CI). Finally, sample sizes varied by exposure and outcome (range: 15,672 to 18,081). Analyses were carried out using Stata/SE 18.

We checked the robustness of findings from the main models in multiple sensitivity analyses. We therefore re-specified models to include additional covariables as potential confounders and, for women, reproductive status. We also determined whether the main association of each transition with changes in healthful eating was independent of other social ties by including them as covariables. Finally, we revised our outcome variable for vegetables by excluding potatoes. Full details are in Supplemental Methods.

## Results

The study averaged 70.3 months (SD 5.5), and the sample mean age was 62 years (SD 9.9) with 59% (cis) women. The socio-demographic characteristics across marital transitions and cohabitation transitions are given in Table 1 for women and Table 2 for men. Across eligible samples, approximately 1 in 5 aging adults developed less healthful eating at FU2. Although most women and men remained partnered (61% and 78%, respectively) or remained co-living (69% and 84%, respectively), we observed adverse changes in close social ties in our sample, particularly among women. Adverse marital transitions (e.g. remaining non-partnered, becoming divorced, or widowed) occurred in 37% of women and 19% of men, and adverse cohabitation transitions (remaining lone-living and becoming lone-living) occurred in 28% of women and 14% of men. Changes in both close social ties were closely related to socio-demographic measures and developing unhealthful vegetable intake (less than once per day) in both women (Table 1) and men (Table 2). Gender-specific descriptives for fruit intake are given in Supplemental Table S3.

**Table 1:** Sample characteristics across transitions in close social ties among aging women with daily vegetable intake at baseline in the Canadian longitudinal study on aging (2011-21)

	Age (years)	Highest education^1^	Highest household income^2^	Home-owner	Urban location	BMI (kg/m^2^)	No chronic condition	Non-smoker	Non-drinker	Not sleep deprived	Non-daily vegetable intake
Marital transitions (*N* = 9206)											
Remained partnered (*n* = 5573)	57.74 (9.03)	4555 (68.8%)	1309 (22.6%)	5307 (94.6%)	4860 (90.8%)	27.44 (5.82)	359 (7.1%)	3023 (49.7%)	582 (14.7%)	3593 (62.3%)	902 (14.8%)
Remained non-partnered (*n* = 2913)	63.33 (10.73)	2186 (56.7%)	74 (1.8%)	2073 (63.0%)	2728 (96.3%)	28.41 (6.48)	87 (4.1%)	1385 (41.8%)	391 (14.5%)	1772 (60.6%)	636 (23.5%)
Became divorced (*n* = 117)	53.44 (6.63)	98 (74.8%)	29 (29.1%)	109 (93.3%)	103 (94.3%)	27.09 (7.03)	6 (6.0%)	62 (58.2%)	13 (13.1%)	72 (64.1%)	24 (20.1%)
Became widowed (*n* = 175)	68.66 (10.04)	127 (51.0%)	10 (3.5%)	149 (78.2%)	160 (94.1%)	26.52 (4.21)	5 (6.5%)	86 (49.3%)	24 (24.6%)	111 (53.8%)	39 (21.0%)
Became partnered (*n* = 160)	56.48 (8.46)	135 (81.7%)	11 (5.2%)	126 (87.9%)	146 (86.9%)	25.96 (5.61)	5 (9.9%)	78 (44.0%)	11 (8.2%)	97 (57.3%)	37 (18.8%)
Uncertain transitions (*n* = 268)	57.95 (10.04)	204 (57.9%)	18 (8.1%)	179 (64.8%)	259 (98.8%)	27.41 (6.03)	17 (5.0%)	94 (30.7%)	24 (18.9%)	172 (65.0%)	62 (15.2%)
Cohabitation transitions (*N* = 9175)											
Remained co-living (*n* = 6342)	57.61 (9.09)	5166 (68.3%)	1355 (20.5%)	5883 (91.6%)	5583 (91.4%)	27.54 (5.92)	407 (7.0%)	3379 (48.5%)	690 (14.7%)	4049 (62.0%)	1090 (16.1%)
Remained lone-living (*n* = 2126)	66.20 (10.11)	1561 (53.5%)	41 (1.2%)	1473 (61.7%)	2003 (96.6%)	27.87 (6.32)	48 (2.4%)	988 (41.8%)	261 (16.4%)	1318 (62.3%)	455 (21.0%)
Became lone-living (*n* = 475)	61.23 (10.46)	375 (59.7%)	38 (7.6%)	385 (71.1%)	422 (93.2%)	27.75 (5.67)	15 (10.3%)	229 (44.1%)	55 (12.4%)	295 (62.1%)	105 (19.0%)
Became co-living (*n* = 232)	62.59 (9.80)	178 (61.3%)	7 (2.5%)	173 (71.9%)	219 (97.9%)	28.23 (6.46)	5 (1.4%)	117 (42.8%)	33 (15.8%)	140 (54.2%)	46 (29.0%)

Descriptive statistics (frequencies (%) and means (SD)) were calculated using CLSA survey inflation weights. All variables were reported at baseline, except for vegetable intake at follow-up 2

^1^The highest education level was post-secondary graduation [university degree]

^2^The highest income was ≥ C$150,000

**Table 2:** Sample characteristics across transitions in close social ties among aging men with daily vegetable intake at baseline in the Canadian longitudinal study on aging (2011-21)

	Age (years)	Highest education^1^	Highest household income^2^	Home-owner	Urban location	BMI (kg/m^2^)	No chronic condition	Non-smoker	Non-drinker	Not sleep deprived	Non-daily vegetable intake
Marital transitions (*N* = 6521)											
Remained partnered (*n* = 5103)	58.91 (9.73)	4314 (70.3%)	1309 (25.3%)	4780 (91.2%)	4574 (94.1%)	28.12 (4.65)	374 (8.9%)	2402 (43.6%)	459 (10.8%)	3377 (63.5%)	1475 (28.3%)
Remained non-partnered (*n* = 948)	60.36 (10.94)	760 (65.1%)	48 (3.6%)	659 (60.3%)	868 (96.5%)	27.62 (5.28)	53 (5.9%)	398 (42.6%)	128 (14.8%)	579 (65.8%)	323 (26.6%)
Became divorced (*n* = 93)	51.92 (6.64)	75 (71.5%)	28 (29.9%)	84 (79.1%)	84 (92.4%)	26.73 (3.54)	4 (8.0%)	41 (38.7%)	13 (12.1%)	50 (70.2%)	41 (41.8%)
Became widowed (*n* = 68)	65.54 (10.09)	52 (51.1%)	11 (15.1%)	59 (80.6%)	64 (98.3%)	27.95 (4.68)	x	26 (27.4%)	11 (21.3%)	50 (82.1%)	30 (26.2%)
Became partnered (*n* = 151)	55.84 (8.74)	128 (78.9%)	26 (14.2%)	132 (86.9%)	136 (96.0%)	26.95 (3.68)	22 (12.1%)	73 (41.4%)	11 (16.8%)	97 (59.5%)	56 (38.0%)
Uncertain transitions (*n* = 158)	59.62 (8.81)	115 (58.5%)	17 (8.2%)	122 (71.4%)	144 (92.1%)	28.47 (4.70)	10 (8.1%)	58 (20.2%)	11 (7.1%)	105 (57.3%)	54 (30.4%)
Cohabitation transitions (*N* = 6497)											
Remained co-living (*n* = 5457)	58.73 (9.69)	4599 (70.0%)	1369 (24.6%)	5074 (90.2%)	4896 (94.2%)	28.11 (4.66)	406 (8.8%)	2562 (42.9%)	488 (10.7%)	3593 (63.2%)	1607 (28.8%)
Remained lone-living (*n* = 729)	61.85 (10.67)	573 (62.1%)	33 (2.6%)	490 (59.4%)	668 (96.0%)	27.86 (5.19)	40 (5.6%)	291 (38.6%)	107 (17.7%)	447 (66.4%)	242 (24.7%)
Became lone-living (*n* = 204)	58.70 (11.3)	158 (64.9%)	28 (15.0%)	161 (69.2%)	188 (95.9%)	26.19 (4.54)	10 (6.0%)	84 (40.5%)	27 (12.3%)	133 (73.9%)	79 (32.4%)
Became co-living (*n* = 107)	57.28 (9.82)	93 (82.3%)	7 (4.7%)	89 (76.3%)	96 (91.6%)	27.12 (3.77)	6 (10.2%)	41 (43.9%)	8 (14.6%)	69 (62.5%)	43 (32.4%)

Descriptive statistics (frequencies (%) and means (SD)) were calculated using CLSA survey inflation weights. All variables were reported at baseline, except for vegetable intake at follow-up 2

^1^The highest education level was post-secondary graduation [university degree]

^2^The highest income was ≥ C$150,000. X, value suppressed due to cell size < 5

### Gender-specific association between changes in close social ties and subsequent reduction in healthful vegetable intake at 6-year follow-up

Table 3 gives the multivariable-adjusted mixed-effects regression results for marital transitions and showed they were prospectively associated with the reduction in healthful vegetable intake at follow-up in a gendered way. In Model D, remaining non-partnered precipitated a change from daily vegetable intake (healthful eating) to less frequent intakes (unhealthful eating) among women (OR 1.21 [95% CI: 1.07, 1.38]), compared to women who remained partnered. Although becoming widowed, becoming partnered, or having uncertain marital transitions also precipitated a change from daily vegetable intake (healthful eating) to less frequent intakes (unhealthful eating) among women in Model A (adjusting for study duration, age, BMI and chronic conditions), results lost significance in Model D (additionally adjusting for SES, health behaviours and provincial confounders). Results for men were more consistent across models: becoming divorced and becoming widowed increased the odds of developing unhealthful (non-daily) intake of vegetables (ORs 1.91 [1.25, 2.92] and 1.91 [1.17, 3.13], respectively), compared to men remaining partnered.

**Table 3:** Gender-specific odds ratios (95% CI) of non-daily vegetable intake associated with marital transitions in the Canadian longitudinal study on aging (2011-21)

Marital transitions	Model A		Model B		Model C		Model D	
	OR	CI95	OR	CI95	OR	CI95	OR	CI95
Women								
Remained partnered	Ref		Ref		Ref		Ref	
Remained non-partnered	1.37***	[1.22, 1.54]	1.21**	[1.07, 1.37]	1.21**	[1.07, 1.38]	1.21**	[1.07, 1.38]
Became divorced	1.39	[0.88, 2.17]	1.39	[0.89, 2.19]	1.38	[0.88, 2.19]	1.38	[0.87, 2.19]
Became widowed	1.46*	[1.02, 2.09]	1.40	[0.99, 2.02]	1.38	[0.96, 2.00]	1.38	[0.96, 2.01]
Became partnered	1.60*	[1.11, 2.32]	1.47*	[1.01, 2.13]	1.45	[0.99, 2.12]	1.45	[0.99, 2.12]
Uncertain transitions	1.51**	[1.12, 2.02]	1.34	[0.99, 1.81]	1.34	[0.99, 1.82]	1.34	[0.99, 1.82]
Men								
Remained partnered	Ref		Ref		Ref		Ref	
Remained non-partnered	1.23**	[1.06, 1.43]	1.07	[0.92, 1.43]	1.08	[0.92, 1.26]	1.08	[0.92, 1.26]
Became divorced	1.92**	[1.27, 2.92]	1.87**	[1.28, 2.95]	1.91**	[1.25, 2.92]	1.91**	[1.25, 2.92]
Became widowed	1.88*	[1.16, 3.06]	1.85*	[1.14, 3.02]	1.91*	[1.17, 3.13]	1.91*	[1.17, 3.13]
Became partnered	1.33	[0.95, 1.86]	1.28	[0.91, 1.80]	1.33	[0.94, 1.87]	1.33	[0.94, 1.87]
Uncertain transitions	1.22	[0.87, 1.71]	1.11	[0.79, 1.56]	1.10	[0.78, 1.55]	1.10	[0.78, 1.54]

Gender-specific odds ratios (95% CIs) obtained by mixed-effects logistic regression with an interaction term (marital transitions and gender) on the sample with daily vegetable intake at baseline conditioning on confounders. Model A: Follow-up time, age, BMI, and chronic condition (*n* = 16,160). Model B: Model A + education, income, home-ownership, and geographic location (*n* = 16,113). Model C: Model B + alcohol, smoking, and sleep deprivation (*n* = 15,727). Model D: Model C + provincial gross domestic product (GDP), % provincial food insecurity, and provincial spending on social protection (*n* = 15,727)

Bold is statistically significant: *p < 0.05; **p < 0.01; ***p < 0.001

Table 4 revealed that specific cohabitation changes were also prospectively associated with the reduction in healthful vegetable intake, with gender differences. Compared to remaining co-living, remaining lone-living or becoming lone-living precipitated a change from daily vegetable intake (healthful eating) to less frequent intakes (unhealthful eating) among women in Model A, but results lost significance in Model D. Results for men were more consistent across models: becoming lone-living and becoming co-living both precipitated a reduction in healthful vegetable intake among men (ORs 1.42 [1.06, 1.91] and 1.55 [1.04, 2.32], respectively), compared to referent (Model D).

**Table 4:** Gender-specific odds ratios (95% CI) of non-daily vegetable intake associated with cohabitation transitions in the Canadian longitudinal study on aging (2011-21)

Cohabitation transitions	Model A		Model B		Model C		Model D	
	OR	CI95	OR	CI95	OR	CI95	OR	CI95
Women								
Remained co-living	Ref		Ref		Ref		Ref	
Remained lone-living	1.25***	[1.11, 1.42]	1.10	[0.96, 1.26]	1.10	[0.96, 1.26]	1.10	[0.96, 1.26]
Became lone-living	1.32*	[1.05, 1.65]	1.24	[0.99, 1.56]	1.25	[0.99, 1.57]	1.25	[0.99, 1.57]
Became co-living	1.20	[0.86, 1.65]	1.04	[0.75, 1.45]	1.01	[0.72, 1.42]	1.01	[0.72, 1.42]
Men								
Remained co-living	Ref		Ref		Ref		Ref	
Remained lone-living	1.16	[0.99, 1.37]	1.00	[0.84, 1.19]	1.01	[0.85, 1.21]	1.01	[0.85, 1.21]
Became lone-living	1.52**	[1.14, 2.03]	1.43*	[1.07, 1.91]	1.42*	[1.06, 1.91]	1.42*	[1.06, 1.91]
Became co-living	1.68*	[1.13, 2.50]	1.53*	[1.03, 2.29]	1.55*	[1.04, 2.32]	1.55*	[1.04, 2.32]

Gender-specific odds ratios (95% CIs) obtained by mixed-effects logistic regression with an interaction term (cohabitation transitions and gender) on the sample with daily vegetable intake at baseline conditioning on confounders. model A: Follow-up time, age, BMI, and chronic condition (*n* = 16,105). model B: model A + education, income, home-ownership, and geographic location (*n* = 16,058). model C: model B + alcohol, smoking, sleep deprivation (*n* = 15,672). model D: model C + provincial gross domestic product (GDP, % provincial food insecurity, and provincial spending on social protection (*n* = 15,672)

Bold is statistically significant: *p < 0.05; **p < 0.01; ***p < 0.001

**Fig. 1:**
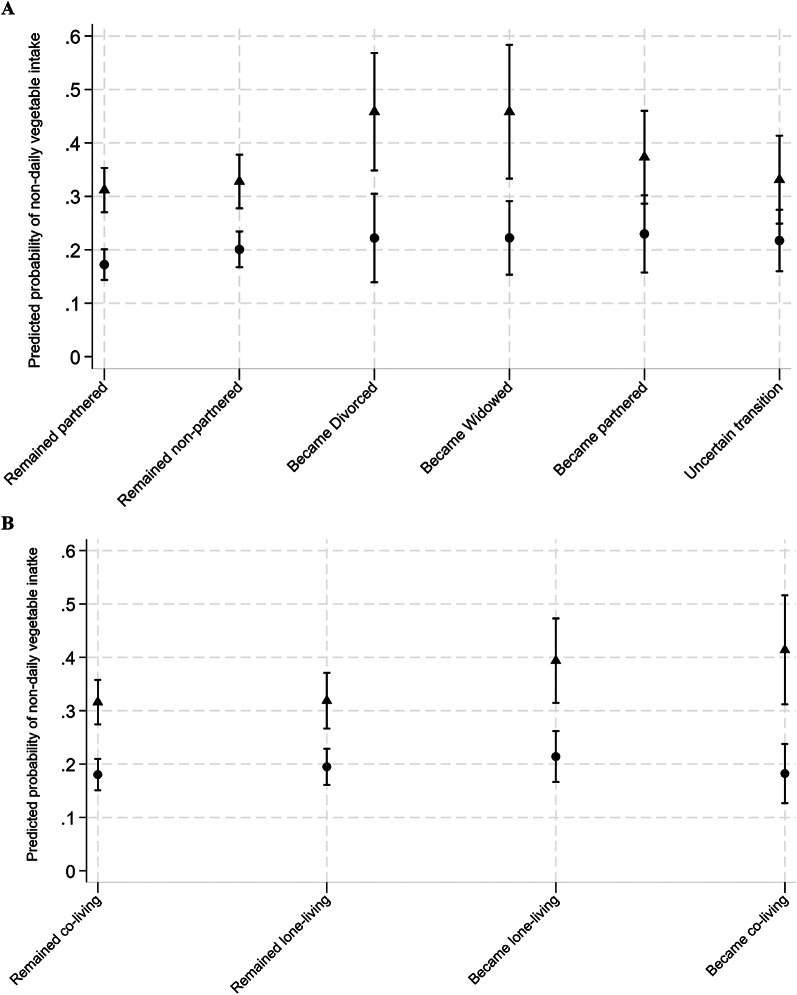
Average predicted probability of non-daily vegetable intake associated with changes in close social ties among aging women and men in the Canadian Longitudinal Study on Aging (2011-21). Triangles represent estimates for men, and circles represent estimates for women, with corresponding 95% confidence intervals. Panel **A**, marital transitions; Panel **B**, cohabitation transitions

The average predicted probability of developing unhealthful vegetable intake varied across different changes in close social ties and differed by gender (Fig. 1). Men generally had higher average probabilities of non-daily vegetable intake than women across all marital and cohabitation transitions.

Sensitivity analyses showed that the odds ratios and average predicted probabilities of non-daily vegetable intake remained largely unchanged after adjusting for other potential confounders (Supplemental Tables S4 and S5). Importantly, the main associations observed for marital transitions and cohabitation transitions were robust to adjustment of other baseline social ties (Supplemental Fig. S3).

### Gender-specific association between changes in close social ties and subsequent reduction in healthful intake of fruits at 6-year follow-up

Marital transitions, as well as cohabitation transitions, were prospectively associated with the reduction in healthful fruit intake at follow-up in a gendered way (Tables 5 and 6). Across all models, becoming divorced precipitated a change from daily fruit intake (healthful eating) to less frequent intakes (unhealthful eating) among women (OR 1.76 [95% CI: 1.16, 2.66] in Model D), compared to remaining partnered. Among men, remaining non-partnered and becoming widowed were associated with a reduction in healthful fruit intake across all models (ORs in Model D were 1.20 [1.03, 1.41] and 1.99 [1.26, 3.15], respectively), compared to referent. In Model D for cohabitation transitions (reference is co-living), women remaining lone-living were less likely to reduce healthful fruit intake (OR 0.86 [0.74, 0.99]) whereas men becoming lone-living or becoming co-living were more likely to reduce healthful fruit intake (ORs 1.48 [1.12, 1.96] and 1.48 [1.00, 2.18], respectively). Although remaining lone-living precipitated a change from daily fruit intake (healthful eating) to less frequent intakes (unhealthful eating) among men in Model A, results lost significance in Model D.

**Table 5:** Gender-specific odds ratios (95% CI) of non-daily fruit intake associated with marital transitions in the Canadian longitudinal study on aging (2011-21)

Marital Transitions	Model A		Model B		Model C		Model D	
	OR	CI95	OR	CI95	OR	CI95	OR	CI95
Women								
Remained partnered	Ref		Ref		Ref		Ref	
Remained non-partnered	1.11	[0.98, 1.26]	1.02	[0.89, 1.63]	1.02	[0.89, 1.17]	1.02	[0.89, 1.17]
Became divorced	1.80**	[1.20, 2.70]	1.76**	[1.17, 2.65]	1.76**	[1.16, 2.66]	1.76**	[1.16, 2.66]
Became widowed	0.85	[0.54, 1.34]	0.83	[0.53, 1.31]	0.83	[0.52, 1.32]	0.83	[0.51, 1.32]
Became partnered	1.20	[0.81, 1.80]	1.15	[0.77, 1.72]	1.07	[0.71, 1.63]	1.08	[0.71, 1.63]
Uncertain transitions	1.37	[1.00, 1.88]	1.25	[0.91, 1.73]	1.27	[0.92, 1.76]	1.27	[0.92, 1.76]
Men								
Remained partnered	Ref		Ref		Ref		Ref	
Remained non-partnered	1.33***	[1.15, 1.54]	1.22*	[1.04, 1.43]	1.20*	[1.03, 1.41]	1.20*	[1.03, 1.41]
Became divorced	1.15	[0.74, 1.81]	1.12	[0.72, 1.76]	1.12	[0.71, 1.75]	1.12	[0.71, 1.75]
Became widowed	2.00**	[1.30, 3.12]	1.98**	[1.27, 3.09]	1.99**	[1.26, 3.14]	1.99**	[1.26, 3.15]
Became partnered	1.32	[0.95, 1.84]	1.28	[0.91, 1.79]	1.29	[0.92, 1.80]	1.29	[0.92, 1.81]
Uncertain transitions	1.26	[0.89, 1.87]	1.16	[0.82, 1.65]	1.09	[0.76, 1.57]	1.10	[0.77, 1.57]

Gender-specific odds ratios (95% CIs) obtained by mixed-effects logistic regression with an interaction term (marital transitions and gender) on the sample with daily fruit intake at baseline conditioning on confounders. Model A: Follow-up time, age, BMI, and chronic condition (*n* = 18,584). Model B: Model A + education, income, home-ownership, and geographic location (*n* = 18,538). Model C: Model B + alcohol, smoking, sleep deprivation (*n* = 18,081). Model D: Model C + provincial gross domestic product (GDP), % provincial food insecurity, and provincial spending on social protection (*n* = 18,081)

Bold is statistically significant: * *p* < 0.05; ** *p* < 0.01

**Table 6:** Gender-specific odds ratios (95% CI) of non-daily fruit intake associated with cohabitation transitions among in the Canadian longitudinal study on aging (2011-21)

Cohabitation transitions	Model A		Model B		Model C		Model D	
	OR	CI95	OR	CI95	OR	CI95	OR	CI95
Women								
Remained co-living	Ref		Ref		Ref		Ref	
Remained lone-living	0.95	[0.82, 1.09]	0.86*	[0.74, 0.99]	0.86*	[0.74, 0.10]	0.86*	[0.74, 0.99]
Became lone-living	1.06	[0.83, 1.37]	1.02	[0.79, 1.32]	1.02	[0.79, 1.31]	1.02	[0.79, 1.31]
Became co-living	1.23	[0.89, 1.70]	1.12	[0.80, 1.56]	1.10	[0.79, 1.55]	1.10	[0.79, 1.55]
Men								
Remained co-living	Ref		Ref		Ref		Ref	
Remained lone-living	1.31**	[1.11, 1.54]	1.16	[0.98, 1.38]	1.16	[0.97, 1.38]	1.16	[0.97, 1.38]
Became lone-living	1.55**	[1.18, 2.05]	1.48**	[1.12, 1.95]	1.47**	[1.11, 1.95]	1.48**	[1.11, 1.96]
Became co-living	1.59*	[1.09, 2.33]	1.54*	[1.05, 2.26]	1.48*	[1.00, 2.18]	1.48*	[1.00, 2.18]

Gender-specific odds ratios (95% CIs) obtained by mixed-effects logistic regression with an interaction term (cohabitation transitions and gender) on the sample with daily fruit intake at baseline conditioning on confounders. Model A: Follow-up time, age, BMI, and chronic condition (n=18,522). Model B: Model A + education, income, home-ownership, and geographic location (n=18,475). Model C: Model B + alcohol, smoking, sleep deprivation (n=18,019). Model D: Model C + provincial gross domestic product (GDP), % provincial food insecurity, and provincial spending on social protection (n=18,019)

Bold is statistically significant: *p<0.05; ** *p*<0.01

**Fig. 2:**
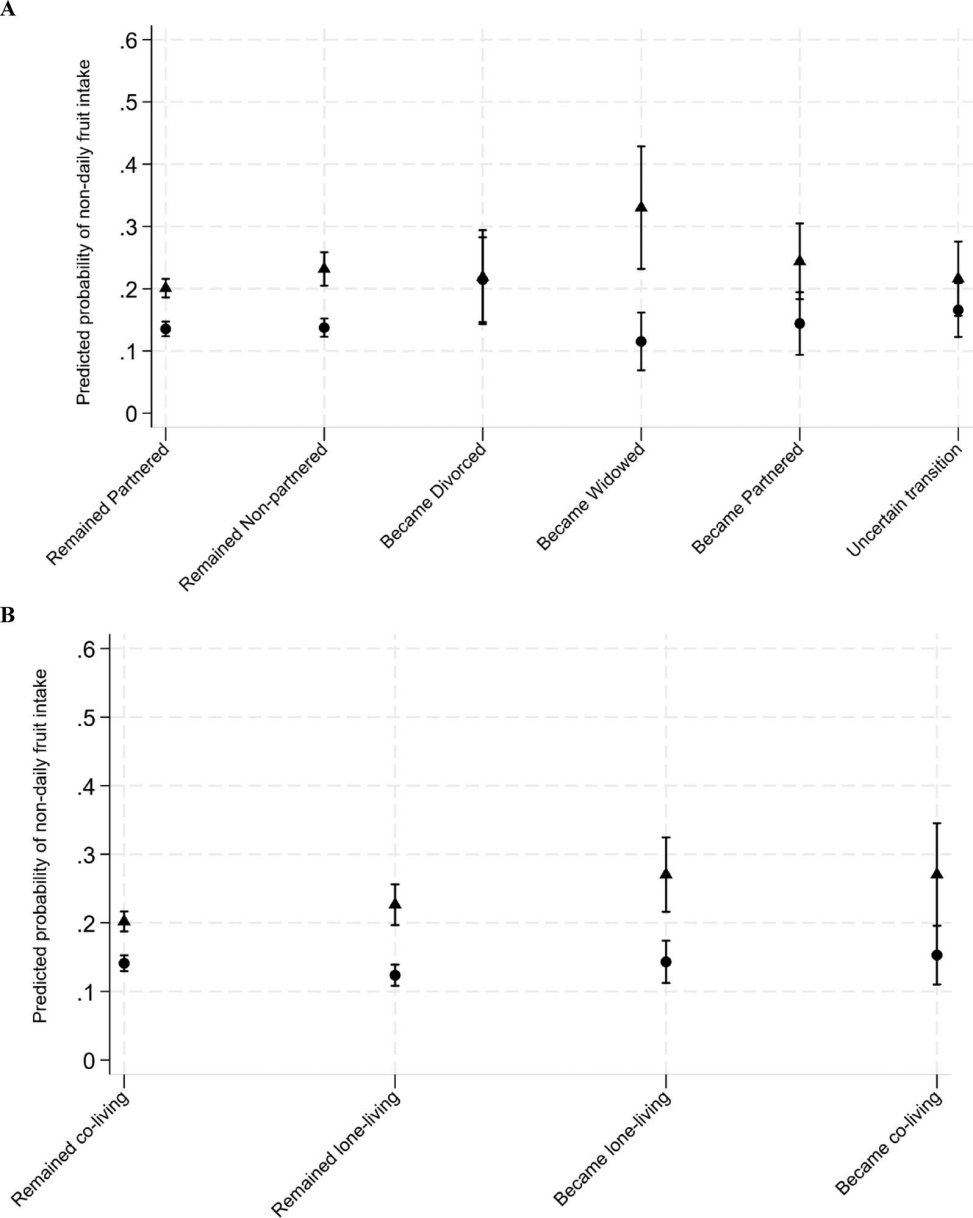
Average predicted probability of non-daily fruit intake associated with changes in close social ties among aging women and men in the Canadian Longitudinal Study on Aging (2011-21). Triangles represent estimates for men, and circles represent estimates for women, with corresponding 95% confidence intervals. Panel**A**, marital transitions; Panel **B**, cohabitation transitions.

The average predicted probability of eating fruit less than one time per day fluctuated across different changes in close social ties in a gendered manner (Fig. 2). For example, becoming widowed had divergent effects for men (increased likelihood) and women (decreased likelihood).

Sensitivity analyses showed that the odds ratios and average predicted probabilities of non-daily fruit intake remained largely unchanged after adjusting for other potential confounders (Supplemental Tables S6 and S7). Importantly, the main associations observed for marital transitions and cohabitation transitions were robust to adjustment of other baseline social ties (Supplemental Fig. S4).

## Discussion

This population-based prospective study investigated whether changes in marital status and living arrangements were associated with changes in healthful eating in middle-aged and older women and men in Canada. Specific adverse changes in close social ties precipitated a change from daily F/V intake (healthful eating) to less frequent intakes (unhealthful eating) in a gendered manner. Consistent with our hypothesis, adverse marital transitions of remaining non-partnered and becoming divorced reduced healthful eating among women. However, women remaining lone-living seemed to be protected from reductions in healthful fruit intake, contrary to our hypothesis. Overall, findings for men were more consistent with our hypotheses that adverse marital transitions (becoming divorced, becoming widowed, remaining non-partnered), and adverse cohabitation transitions (becoming lone-living) precipitated a reduction in healthful eating. Surprisingly, we also found that women entering marriage and men becoming co-living appeared to be at risk of unhealthful eating. Finally, an important research finding is that the prospective associations of both marital and cohabitation transitions with changes in healthful eating were independent of other social ties. This indicates that dietary alterations resulting from marital transitions are not contingent upon changes in living arrangement (or vice versa) and reinforces the unique and distinct role of different types of close social ties for health-related outcomes for women and men [24, 60].

### Relevance to previous research

Reviews indicate that having a social tie is linked to greater survival [3, 61], and nutritional epidemiologic research shows different types of social ties influence diet quality [62], and specifically affect FV consumption [24]. Married older adults consume greater amounts of FV than non-married counterparts [63], whereas widowed older adults tend to have poorer diet quality than non-widowed counterparts [26]. Living arrangements and social contacts are independently associated with diet quality among older adults [24, 27, 64]. Specifically, reduced social participation, lower social support and social isolation are all associated with greater nutritional risk [65] and lower FV consumption [66]. Only a handful of longitudinal studies exist to compare our findings on specific alterations in close social ties and diet quality and previous research lack temporality between exposures and outcomes or rarely report sex-disaggregated data [32, 34, 36]. Existing prospective evidence only considers marital transitions [29], with no prior nutrition research on the dietary effects of cohabitation transitions regardless of marital status. Moreover, cross-sectional studies in nutrition that assess lone-living [66, 67] typically incorporate lone-living with other social ties into a summary index of ‘social isolation’ as a broader concept. This methodological approach obscures the unique influence of each type of close social tie as a distinct determinant of diet. Our prospective study of aging Canadians is the first, to our knowledge, to examine and contrast changes in two types of close social ties as potential determinants of diet quality and to provide disaggregated data on aging women.

Our study corroborates the limited longitudinal research showing gender differences in the relationship between marital transitions and changes in healthful eating indicators. Consistent with our findings, Vinther and colleagues showed that British women who became divorced or stayed non-partnered had decreases in vegetable variety, while British men who had different changes in marital status (e.g. became widowed, became divorced, and stayed non-partnered) reduced their quantity and/or variety of both fruits and vegetables [32]. Similarly, in US prospective studies of marital transitions and diet, vegetable intake reduced among women who divorced, became widowed or stayed non-partnered [31] and diet quality declined among men who underwent spousal loss [30]. There is conflicting research on the role of divorce for diet quality among women [68]. Moreover, previous research suggests that widowhood had similar adverse dietary effects for men and women but declines are greater for men. Women in Japan who became widowed decreased their vegetable consumption but men showed a greater decline [34]. In a French study, 38% of men and 22% of women decreased their daily intake of vegetables after the death of a spouse [36]. Other studies examining FV combined reported short- and long-term (1 to 10 years) negative consequences of both widowhood and divorce [33, 35].

Our work suggests that specific types of marital dissolution have detrimental effects on healthful eating that are gendered. We found that entering both widowhood and divorce had negative effects on the frequency of vegetable intake among men only. In addition, entering widowhood also reduced the daily intake of fruit among men, whereas becoming divorced/separated reduced the daily intake of fruit among women. Reductions in diet quality from widowhood among older adults is consistently reported in the literature [69]. The end of a long-term relationship and the resulting lack of companionship during meals can often lead to feelings of loneliness and a reduced interest in food-related activities such as meal planning, grocery shopping, and cooking [26]. Men in particular who lose their spouses may struggle more with food/meal preparation and be more vulnerable to consequent declines in FV consumption than women in similar situations given that men are historically less involved in activities of food preparation [70]. Traditional gender norms ascribe the role of food procurer and food budgeting to men, whereas women are ascribed with the role of caregiver (including cooking) in a marriage [71]. Studies on marriage and health highlight gender differences in marital dynamics, with women typically investing more effort in maintaining their spouse’s health than men which includes preparing vegetables and cooking healthful meals [72]. Qualitative nutrition research on gender and widowhood identifies a two-staged process of women’s transformation of their personal food systems, whereby the loss of regular shared meals first leads to women adapting to new eating patterns followed by a shift in dietary focus away from the needs of the couple to fulfill their individual nutritional requirements [73]. Thus, evidence suggests that widowhood is less likely to negatively affect women’s vegetable intake as found in this study. Women who experience divorce/separation, however, were found to be at risk of reducing healthful eating, which might be attributed to the higher cost of fruit in Canada [74] combined with women’s greater financial constraint from no longer having access to their husband’s pensions, unlike widowed women [75].

Our data also showed that persistent lack of partnership precipitated reduction in daily vegetable intake among women but daily fruit intake among men. Others have also examined this category of marital transition [30–32, 34, 35] and reported adverse consequences mainly for vegetable intake [31, 32]. As this study could not account for marital status prior to cohort entry, it is possible that adverse dietary effects of remaining non-partnered may in fact be the result of a lagged effect of marital loss prior to cohort entry. If this were the case, then we might expect to also see adverse changes in daily F/V intake among women and men who becoming divorced or widowed during the study period. However, we did not see this same pattern for both types of marital loss among men across all models and, although women who had marital loss appeared to have higher odds of non-daily vegetable intake, these associations were non-significant. Moreover, only women becoming divorced significantly reduced their daily fruit intake while women becoming widowed were protected from adverse dietary changes and women remaining non-partnered had no change in fruit intake. Thus, the dietary change associated with remaining non-partnered is likely to be distinct from behaviour change associated with marital loss. Moreover, it is worth noting that the interpretation of this finding is complicated by the heterogeneity of this ‘transition’ category as it also includes individuals reporting being ‘single/never married’ at cohort entry through to FU1, similar to other studies [32, 34]. Future research should consider short-term and long-term (lagged) effects on healthful eating of remaining non-partnered while accounting for timing and duration using multiple waves of cohort data.

Surprisingly, our results suggest that entering an intimate partnership (becoming married/living as married) might also reduce daily intakes of vegetables among women, meaning we saw a gender-specific *higher* likelihood of non-daily intake of vegetable at follow-up. These results contradict the longstanding concept that marriage provides protective benefits for longevity through health-promoting behaviours—a concept that has endured for over a century with origins from Durkheim’s seminal research on suicide [76]. Previous nutrition research has reported that vegetable intake improves after re-marriage among aging men [30] and aging women [31]. Yet, some longitudinal research shows no association between entering marriage and diet quality [32, 34, 35], or relevant studies do not evaluate the dietary effects of transitioning into marriage [33, 36]. It appears that more research is needed to understand whether and to what extent transitions into a partnership might positively or negatively influence F/V intake in the older population, and to understand whether the dietary effects of this presumably favourable marital transition are gendered. In general, marital loss represents the reduction of social resources and staying non-partnered constitutes a chronic absence of social resources.

Our prospective study adds novel empirical evidence on changes in cohabitation associated with two indicators of healthful eating, with a gender perspective. We found negative dietary effects associated with becoming both lone-living and co-living among men, but protective effects from remaining lone-living among women. This finding remained after also considering marital status, social network size and social participation, and indicates that cohabitation changes are an independent risk factor for healthful eating. Prior research is limited to cross-sectional studies of living arrangements and dietary intake [28, 40], and typically assumes that lone-living is synonymous with lack of a spouse and having a spouse is assumed to indicate co-living [77], which does not capture complex situations of being married but living alone. Older US adults who live alone with no nearby children or friends had the lowest FV intake, a finding that was stronger among men [28]. Although that study showed the presence of nearby friends or family appeared to mitigate the negative influence of lone-living on FV intake [28]. Having children and friends nearby might increase the number of social gatherings and prevent lone-living older adults from eating alone by motivating cooking and eating [78].

Lone-living has also been associated with lower consumption of cereals, meat, eggs, and dairy products, which reduces overall diet quality and may reflect lower meal frequency than older adults living with grandchildren, adult children, or someone else [79]. Indeed, different types of living arrangement (lone-living, living with a spouse, living with a non-spouse) as well as income have been associated with diet quality among older US adults, particularly among older men [40]. Specifically, men living with a spouse consistently had better diets than men living alone or with a non-spouse; moreover, low-income, lone-living men had the worse diets [40]. That study emphasised that simply living with someone is not the primary factor for a better diet; instead, having a spouse appeared more important for older men [40]. By contrast, other research suggests that men do not improve their diets within the first year of spousal cohabitation [80]. This corroborates our surprising finding that men who become co-living were more likely to not meet the recommended daily consumption of F/V daily as part of healthful eating. More research is needed to understand why becoming co-living in later life may be a separate risk factor for reduced frequency of intakes of F/V among older men. For women, our finding of a protective association between remaining lone-living and daily fruit intake may be explained by the fact that a person living alone does not have to consider the food preferences and needs of others [81].

Previous cross-sectional research and our longitudinal results underscore the complex interplay of both gender and close social ties as potential determinants of food choices and healthful eating among older adults. Gender roles, relations and behaviours have a significant influence on an older person’s food choices: women generally choose healthier foods and maintain regular meals, while men prefer specific tastes and meal behaviours [82]. Indeed, the known differences in dietary patterns between men and women is driven by cultural norms, social factors and biological requirements [82]. Men’s diets appear to be more strongly influenced by the presence of a wife whose gendered role is typically to plan and prepare family meals. Yet, our findings reveal a more nuanced picture of gender differences in living arrangement and diet. It was not clear that becoming co-living (independent of marital status) or becoming partnered (independent of cohabitation) was associated with better healthful eating habits in aging women or men. In fact, these changes were linked to less healthful eating. Nevertheless, household composition is an important factor to capture when studying the role of cohabitation changes for diet and there are likely synergistic effects between changes in both marital status and living arrangement that have gendered implications for F/V intakes that require further research. Additionally, future studies should consider whether the dietary effects of specific changes in close social ties vary across age subgroups given age-related variation in the adverse changes in close social ties among Canada’s older population [50].

## Limitations and strengths

Self-reported measures of both the exposure and outcome may introduce misclassification bias in our study’s results. Some CLSA participants may be classified in the wrong transition category due to recall errors about F/V frequency of intake or misunderstanding of the marital status ‘single’. Potential recall bias would be similar across individuals and is mitigated by our analysis of daily consumption as a pattern rather than absolute intakes. Any misclassification of marital transitions from incorrect information on a participant’s ‘single’ status is expected to be the same across the outcome groups and non-differential; thus, results would be biased towards the null. Lack of information on marital status prior to CLSA entry could also be a source of misclassification bias and limited the ability to adequately capture duration of marital transitions over the study period that could affect causal inference about the timing of changes in healthful eating. There may also be selection bias in our sample due to dietary data at follow-up 2 being collected during the COVID-19 pandemic, which limited the number of observations to participants who could access the Data Collection Sites. This study is also subject to both residual confounding (poor measurement of included covariables) and unmeasured confounding (lack of variables for adjustment, e.g., the CLSA did not comprehensively collect dietary data to calculate total energy intake, Kcal); moreover, we note that BMI is a poor proxy for unavailable information on total energy intake despite its common use in nutritional epidemiology research. There is also unmeasured confounding from dietary data that are absent from the SDQ (serving sizes, and food preparation skills) and missing data on the quality of marriage. The use of the SDQ among CLSA participants may also raise reliability concerns. Although the SDQ showed reasonable accuracy for ranking the frequency of selected foods and nutrients (*r* = 0.45 for fruits and vegetables) in the Québec Longitudinal Study on Nutrition and Successful Aging (NuAge) study [48], the 2018 validation study by Gilsing and colleagues [83] did not evaluate its ability to rank fruit and vegetable intake among CLSA participants. Finally, external validity of this study is limited by the nature of the population-based cohort which recruited Canadians who are predominantly White (96%), cisgender (99%) and heteronormative (98%). Our findings can therefore not be generalized to more diverse populations or younger adults.

Yet, this study has numerous strengths. These include: a large sample size, two indicators of healthful eating and two measures of close social ties, adjustment for multiple known confounders, including broader socio-political differences across provinces, advanced statistical techniques to account for nested data and a gender perspective. Of significance, the exposure variable for marital transitions distinguished between different types of transitions out of partnership (becoming widowed versus becoming divorced/separated) which is an important construct that previous research lacks; our exposure variable also included an uncertain category to reduce misclassification bias. These distinct categories of change in marital status are particularly critical for revealing notable gender differences as found in this study which can help inform more targeted strategies for healthful aging. And, finally, this study used an analytic strategy that enabled a more robust causal-effect estimation [84] by ensuring temporality between changes in exposures and outcomes among a sample of participants free of the outcome at baseline.

## Conclusions

Results showed that adverse changes in close social ties can increase the risk of less healthful eating by reducing the frequency of fruit or vegetables intakes among aging Canadians, and that the negative dietary effects are gendered. Public health nutrition and healthful aging strategies should consider fostering supportive social environments as people age, particularly for older men. Future research on potential mediators, such as the quality of social ties, is warranted.

## Supplementary Information

Below is the link to the electronic supplementary material.

Supplementary Material 1

## Data Availability

Data are available from the Canadian Longitudinal Study on Aging (www.clsa-elcv.ca) for researchers who meet the criteria for access to de-identified CLSA data.

## Abbreviations

CLSA: Canadian Longitudinal Study on Aging
F/V: Fruit or vegetable
FV: Fruit and vegetable
FU: Follow–up
BL: Baseline
SES: Socioeconomic status
BMI: Body mass index
SDQ: Short dietary questionnaire
FFQ: Food frequency questionnaire
GDP: Gross domestic product
OR: Odds ratios
95% CI: 95% Confidence Intervals
