# Supplementary material for: Adverse changes in close social ties reduce fruit and vegetable intake in aging adults: a prospective gender-sensitive study of the Canadian longitudinal study on aging (CLSA)
Source: Int J Behav Nutr Phys Act. 2025 Aug 13;22:109. doi: 10.1186/s12966-025-01807-7 (PMC12345031; doi:10.1186/s12966-025-01807-7)
Supplement: Supplementary file 1 — Supplementary Material 1 [file 12966_2025_1807_MOESM1_ESM.docx]

**Adverse changes in close social ties reduce fruit and vegetable intake in aging adults: a prospective gender-sensitive study of the Canadian Longitudinal Study on Aging (CLSA)**

Sanaz Mehranfar, MSc, Gilciane Ceolin, Ph.D., Rana Madani Civi, MSc, Heather Keller, Ph.D., RD, Rachel A. Murphy, Ph.D., Tamara R. Cohen, Ph.D., RD, Annalijn I. Conklin, M.P.H, Ph.D. (Cantab)

**Table of contents**

**Supplemental Methods**

Study population

Changes in fruit or vegetable intake

Transitions in marital status

Covariables

Sensitivity analyses

**Supplemental Results**

**Supplemental** **Table S1.** Coding process of mock examples of dietary data from the CLSA Short Diet Questionnaire.

**Supplemental Table S2.** Changes in marital status between baseline and follow-up 1 in the Canadian Longitudinal Study on Aging classified as an uncertain transition.

**Supplemental Table S3.** Sample characteristics across transitions in close social ties among aging women and men with daily fruit intake at baseline in the Canadian Longitudinal Study on Aging (2011-21).

**Supplemental Table S4.** Sensitivity analysis of the associations between marital transitions and non-daily vegetable intake among aging women and men in the Canadian Longitudinal Study on Aging (2011-21).

**Supplemental Table S5.** Sensitivity analysis of the associations between cohabitation transitions and non-daily vegetable intake among aging women and men in the Canadian Longitudinal Study on Aging (2011-21).

**Supplemental Table S6.** Sensitivity analysis of the associations between marital transitions and non-daily fruit intake among aging women and men in the Canadian Longitudinal Study on Aging (2011-21).

**Supplemental Table S7.** Sensitivity analysis of the associations between cohabitation transitions and non-daily fruit intake among aging women and men in the Canadian Longitudinal Study on Aging (2011-21).

**Supplemental Figure S1.** Flow diagram of the process of sample selection from the Canadian Longitudinal Study on Aging (2011-21).

**Supplemental Figure S2.** Directed Acyclic Graph (DAG) for marital transitions (a) and cohabitation transitions (b) and fruit and vegetable intake.

**Supplemental Figure S3.** Average predicted probability of non-daily vegetable intake associated with changes in close social ties among aging women and men in the Canadian Longitudinal Study on Aging (2011-21), independent of other social ties.

**Supplemental Figure S4.** Average predicted probability of non-daily fruit intake for sensitivity analysis of changes in close social ties while adjusting for other social ties among aging women and men in the Canadian Longitudinal Study on Aging (2011-21).

**Supplemental References**

**Supplemental Methods**

***Study population***

The Canadian Longitudinal Study on Aging (CLSA) Comprehensive Cohort comprised self-reported information gathered through interviews and anthropometric measurements obtained from English- or French-speaking individuals residing within a 25-50 km of 11 specified data collection sites. Those excluded from the cohort included residents of the three territories, federal First Nations reserves and other First Nations settlements in the provinces, remote regions, full-time members of the Canadian Forces, and those living in institutions or with cognitive impairment at the time of recruitment (1, 2). To address the under-representation of individuals with lower education levels and socioeconomic status in population-based studies, special efforts were undertaken to oversample specific areas identified through census data (3). The detailed CLSA study design is available elsewhere (1-3). **Supplemental Figure S1**. shows the flow diagram of the process of sample selection from the CLSA (2011-21).

| **A**  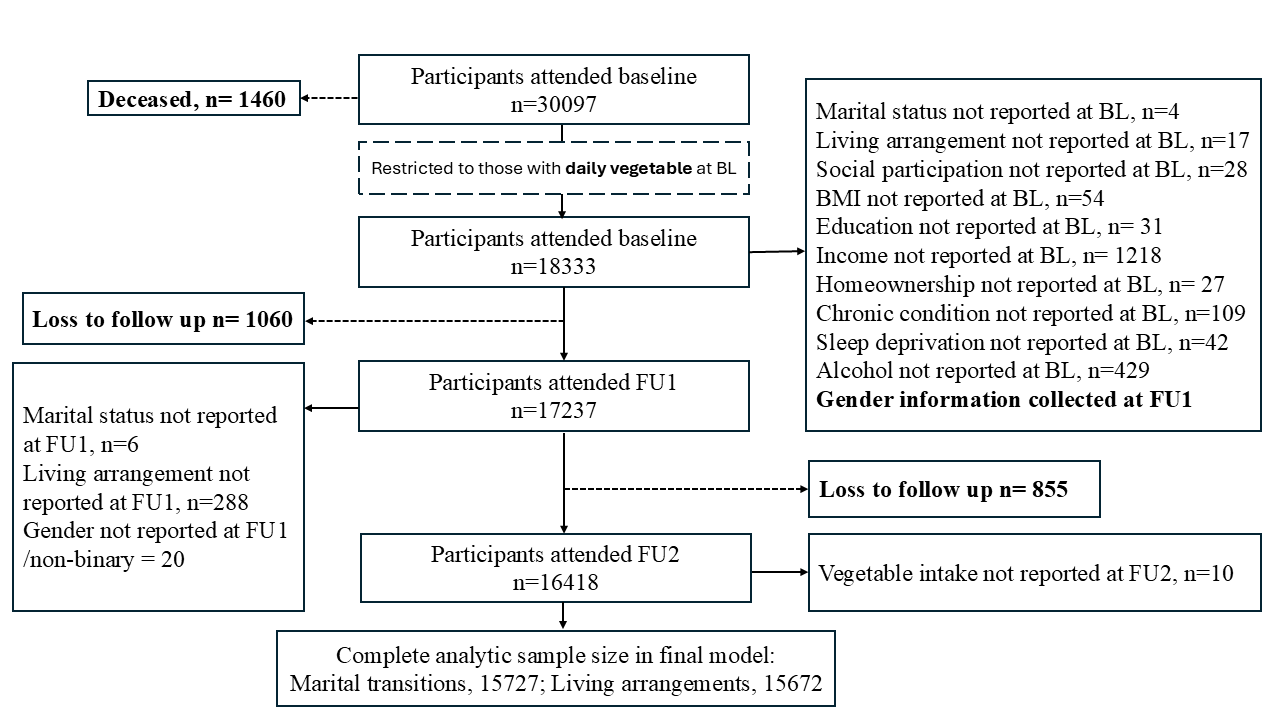 |
| --- |
| **B**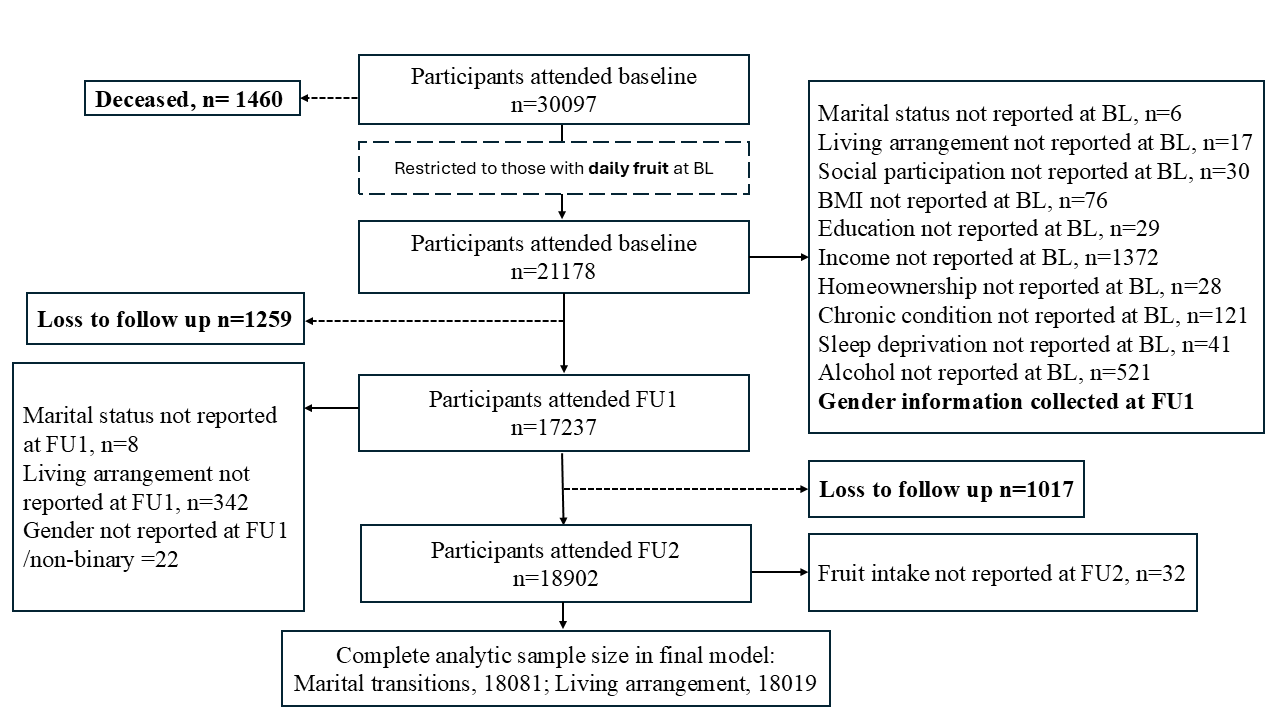 |

**Supplemental Figure S1**. Flow diagram of the process of sample selection from the CLSA (2011-21). Panel A, analytic sample for vegetable intake; Panel B, analytic sample for fruit intake.

***Changes in fruit or vegetable intake***

Dietary information was collected using the same 36-item Short Diet Questionnaire (SDQ) that was developed and used in the CLSA to assess usual consumption frequencies (last 12 months) of key nutrients and foods of importance for health promotion and chronic disease prevention in younger and older adults (4). The SDQ has been tested for use in community-dwelling older adults in a subsample of the Québec Longitudinal Study on Nutrition and Successful Aging (NuAge) Study (4). The SDQ has been validated relative to three 24-h diet recalls and has been shown to be a reasonable approach to obtain usual frequencies of food sources of fats, regular and low-fat food choices, fibre, calcium, vitamin D, whole grains, calcium fortified foods and beverages, and fruits and vegetables (4). CLSA responses for consumption of food items were daily, weekly, monthly, or yearly, followed by another question of “how many times” for that frequency response (e.g., twice a day, three times a week, once a month). CLSA converted all SDQ responses into times per day that we dichotomized for fruits (0 = less than daily, 1 = daily), green vegetables (0 = less than daily, 1 = daily), potatoes (0 = less than daily, 1 = daily), carrots (0 = less than daily, 1 = daily), and other vegetables (0 = less than daily, 1 = daily) (details of coding method in **Supplemental Table S1**). Fruit intake data did not include fruit juices as Canada's Food Guide classifies fruit juices as sweetened beverages (5). We followed the National Cancer Institute Dietary Screener Questionnaire in the US NHANES 2009–2010 method (6) to exclude outliers in daily intake (more than 8 times/day for fruit, 5 times/day for vegetables including carrots, green vegetables, and others, and 3 times/day for potatoes) before constructing binary F/V variables at both BL (to restrict the sample) and FU2 (to assess outcome). Additionally, the guideline recommending at least 400 grams of FV daily, equating to five or more servings, assumes that once per day equals one serving per day. However, this assumption does not apply to CLSA data because the questionnaire did not assess serving sizes, and participants were not informed about typical serving sizes.

**Supplemental** **Table S1. Coding process of mock examples of dietary data from the CLSA Short Diet Questionnaire.**

| **Entity ID** | **Items** | **Per year/**  **Never** | **Per month** | **Per week** | **Per day** | **CLSA data (times per day)** | **Daily or non-daily** |
| --- | --- | --- | --- | --- | --- | --- | --- |
| #1 | Fruit |  |  | 7 |  | 1 | Daily |
| #1 | Green salad |  |  |  | 2 | 2 | Daily |
| #1 | Potatoes |  | 15 |  |  | 0.5 | Non-daily |
| #1 | Carrots |  |  | 5 |  | 0.71 | Non-daily |
| #1 | Other vegetables |  |  | 14 |  | 2 | Daily |
| #2 | Fruit |  |  |  | 5 | 5 | Daily |
| #2 | Green salad |  | 25 |  |  | 0.83 | Non-daily |
| #2 | Potatoes |  |  | 2 |  | 0.29 | Non-daily |
| #2 | Carrots | 0 |  |  |  | 0 | Non-daily |
| #2 | Other vegetables |  |  | 20 |  | 2.86 | Daily |

CLSA question was “How often do you usually eat each food group? For example, twice a day, three times a week, once a month?". CLSA converted frequency responses to times per day using 7 as the denominator for weekly intake and 30 for monthly.

***Transitions in marital status***

Some responses for the marital status question at baseline and follow-up 1 led to unclear or improbably marital transitions (**Supplemental Tables S2**). For example, it is not possible to be single (which means never married by definition) at baseline and then divorced or separated at follow-up 1 as this would imply becoming married very shortly after the baseline assessment and then almost immediately separating for a minimum of 12 months by Canadian law and undergoing a divorce in less than 12 months which is improbable. Similarly, it is not possible to transition from a married or living as married status to a single status which is defined as never married. This example suggests that respondents may be misreporting a single status when in fact they are separated or divorced. For these reasons, we chose to classified these unclear or implausible transitions as “uncertain” following similar research (7, 8).

**Supplemental Table S2. Changes in marital status between baseline and follow-up 1 in the Canadian Longitudinal Study on Aging classified as an uncertain transition.**

| **Baseline**  **(2011-2015)** | **Follow-up 1**  **(2015-2018)** | **N**  **(vegetable intake)** | **N**  **(fruit intake)** | **Classification** |
| --- | --- | --- | --- | --- |
| Married/partnered | Single (never married) | 117 | 119 | Uncertain transition |
| Single (never married) | Divorced/separated | 71 | 81 | Uncertain transition |
| Single (never married) | Widowed | 13 | 15 | Uncertain transition |
| Widowed | Single (never married) | 21 | 27 | Uncertain transition |
| Widowed | Divorced/separated | 23 | 26 | Uncertain transition |
| Divorced/separated | Single | 149 | 162 | Uncertain transition |
| Divorced/separated | Widowed | 32 | 36 | Uncertain transition |

***Covariables***

**Supplemental Figure S2** shows the Directed Acyclic Graph (DAG) we created with DAGitty software (version 3.0; Nijmegen, GE, The Netherlands) (9), to identify theoretical, plausible, and known confounders (10-18). Relevant CLSA covariables at BL included: 1) study duration and biological factors (age, body mass index (BMI) as a proxy of energy intake, and having one or more chronic conditions including depression); 2) socioeconomic status indicators (SES) (education (post-secondary graduation [university degree], some post-secondary education, secondary school graduation [high school diploma], below secondary school graduation [less than high school diploma]), household income (≥C$150,000, ≥C$100,000 to C$149,999, ≥C$50,000 to C$99,999, ≥C$20,000 to C$49,999, <C$20,000, don’t know/refuse), wealth (home owner, renter) and rural/urban location); 3) behavioural factors (alcohol intake frequency, ever-smoker, and at least 7 hr/day of sleep); and 4) province-level covariables (provincial gross domestic product (GDP), provincial expenditure on social protection, and provincial food insecurity) were created based on the average recruitment date of participants in each province. Missing and refused responses are common for income variables and thus these cases were retained for analysis. Notably, self-reported home-ownership is a common SES indicator as it is a good measure of wealth in older populations (18).

| A  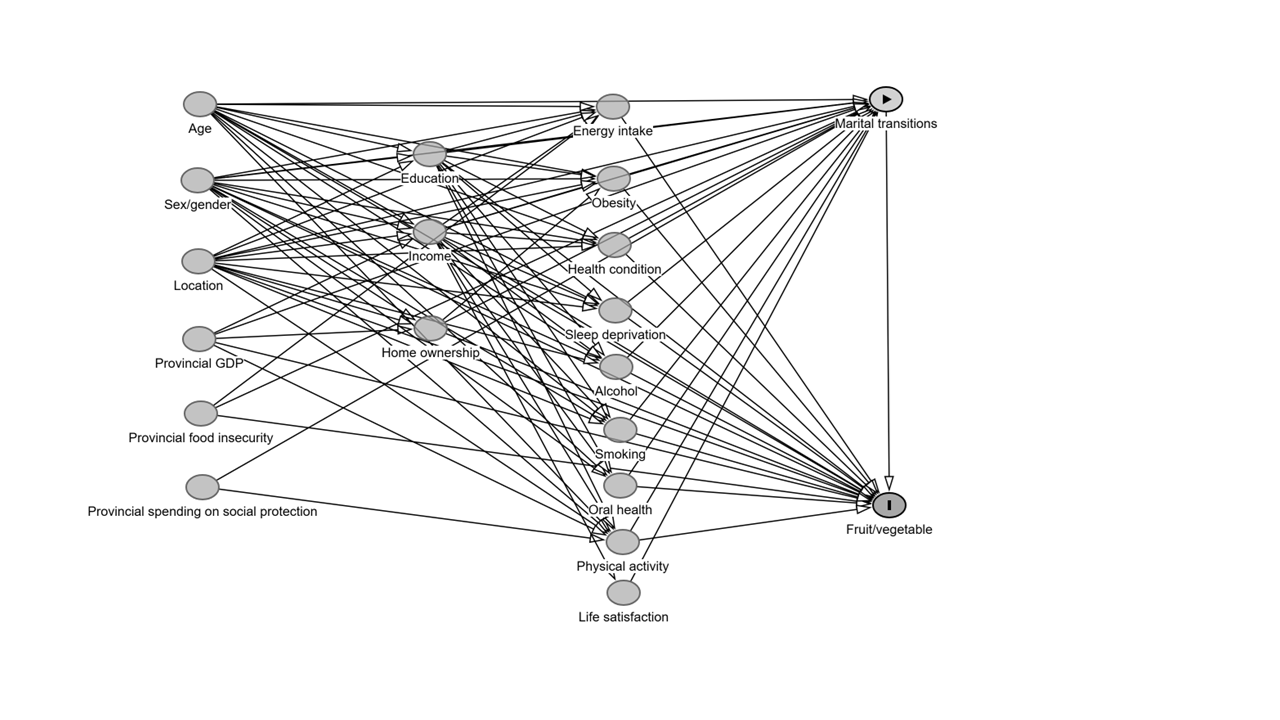 |
| --- |
| B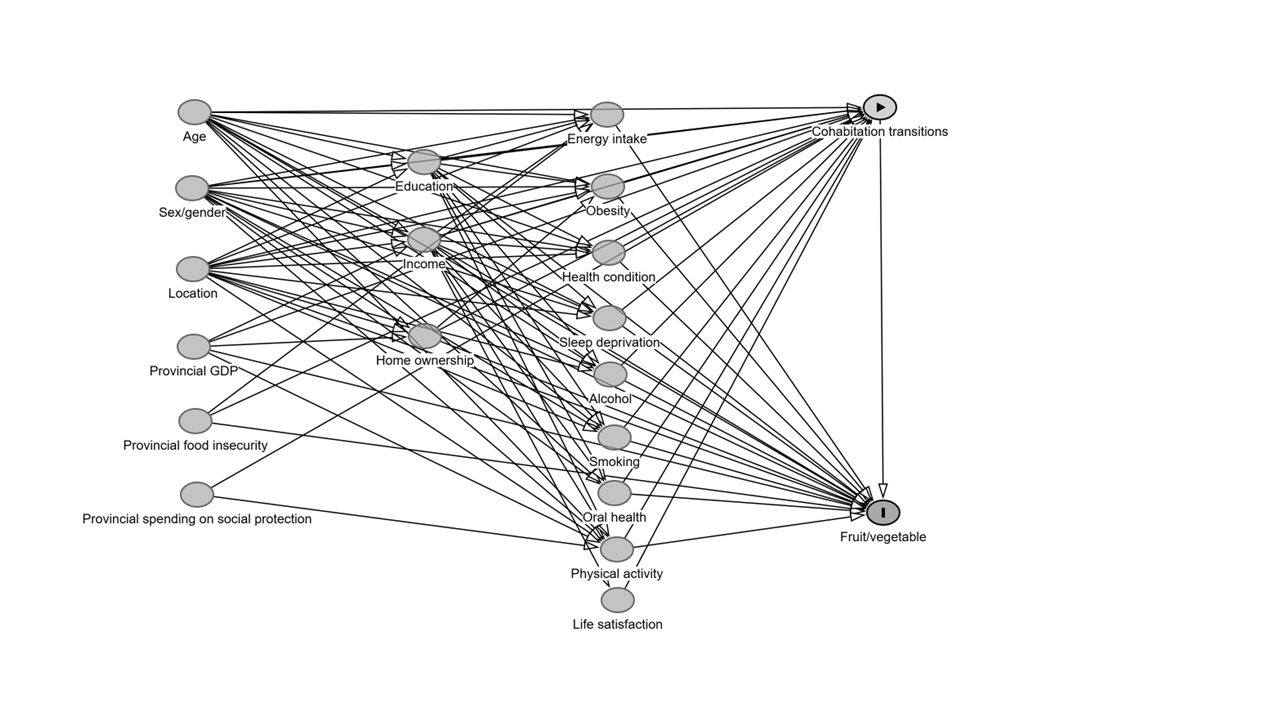 |

**Supplemental Figure S2. Directed Acyclic Graph (DAG) for marital transitions (a) and cohabitation transitions (b) and fruit and vegetable intake.** The minimum set of adjustments covariates for confounding was constructed based on the associations described in the literature to test the association between close social tie transitions and F/V intake. The exposures are marital transitions or cohabitation changes, and the outcome variable is F/V intake. Each variable is represented by the circles; the arrows represent the causal association between each variable. GDP, Gross domestic Product. The exposures are marital transitions and cohabitation changes (play symbol) and the outcome variable is the fruit/vegetable (stop symbol).

***Sensitivity analyses***

Sensitivity analyses of main results were further conditioned on oral health (19), physical activity, life satisfaction and, for women, reproductive variables (pregnancy, menopausal status, and hormone replacement therapy usage). We also considered the independent effects of each transition variable and additionally adjusted final models for other BL social relationships (e.g. living arrangement (lone-living vs. co-living), social participation (number of monthly social activities; score range from 0 to 8), and social network size (1 to 573 known contacts) for marital transition model). Finally, we excluded potato from the vegetable outcome and performed a sensitivity analysis of a recoded vegetable variable based on daily intakes of only three items (carrots, green, and other vegetables).

**Supplemental Results**

**Supplemental Table S3.** **Sample characteristics across transitions in close social ties among aging women and men with daily fruit intake at baseline in the Canadian Longitudinal Study on Aging (2011-21).**

| **Changes in close social ties** | **Age (years)** | **Highest education level^1^** | **Highest income level^2^** | **Home-owner** | **Urban location** | **BMI (kg/m^2^)** | **No chronic condition** | **Non-smoker** | **Non-drinker** | **Not**  **sleep deprived** | **Non-daily fruit intake at FU2** |
| --- | --- | --- | --- | --- | --- | --- | --- | --- | --- | --- | --- |
| **Women** | | | | | | | | | | | |
| **Marital transitions** |  |  |  |  |  |  |  |  |  |  |  |
| Remained partnered (n=6015) | 57.73 (9.04) | 4881 (68.4%) | 1399 (22.6%) | 5709 (94.8%) | 5280 (91.1%) | 27.40 (5.79) | 395 (7.7%) | 3304 (50.1%) | 627 (14.1%) | 3844 (62.3%) | 808 (15.5%) |
| Remained non-partnered (n=3371) | 63.56 (10.69) | 2552 (58.9%) | 78 (1.9%) | 2410 (64.4%) | 3178 (96.8%) | 28.65 (6.38) | 91  (3.8%) | 1655 (44.6%) | 454 (14.6%) | 2054 (60.5%) | 480 (15.5%) |
| Became divorced (n=134) | 53.57 (6.65) | 109 (75.3%) | 32 (27.2%) | 121 (91.2%) | 118 (94.3%) | 27.76 (7.02) | 8  (6.7%) | 72 (52.2%) | 18 (13.6%) | 83 (66.6%) | 31 (16.0%) |
| Became widowed (n=205) | 68.84 (9.93) | 151 (53.2%) | 11 (3.3%) | 178 (86.4%) | 187 (93.6%) | 26.8 (4.76) | 6  (3.9%) | 103 (49.9%) | 27 (21.5%) | 126 (56.2%) | 21 (15.7%) |
| Became partnered (n=185) | 56.96 (8.48) | 157 (77.2%) | 12 (5.6%) | 142 (83.4%) | 172 (87.6%) | 26.95 (6.57) | 7  (9.7%) | 95 (47.2%) | 16 (9.1%) | 113 (56.2%) | 28 (12.9%) |
| Uncertain transitions (n=282) | 58.40 (9.67) | 219 (64.9%) | 16 (8.1%) | 189 (63.6%) | 273 (98.1%) | 28.13 (5.66) | 17  (4.5%) | 110 (36.6%) | 27 (12.3%) | 181 (61.4%) | 50 (21.2%) |
| **Cohabitation transitions** | | | | | | | | | | | |
| Remained co-living (n=6906) | 57.62 (9.12) | 6906 (78.0%) | 1450 (20.4%) | 6369 (78.0%) | 6121 (91.8%) | 27.54 (5.87) | 447 (7.3%) | 3751 (49.5%) | 763 (14.4%) | 4372 (61.9%) | 978 (15.9%) |
| Remained lone-living (n=2457) | 66.59 (9.79) | 1821 (56.7%) | 43 (1.3%) | 1719 (63.1%) | 2329 (96.8%) | 28.39 (6.26) | 51  (2.7%) | 1178 (44.6%) | 302 (14.6%) | 1523 (61.3%) | 310 (13.5%) |
| Became lone-living (n=526) | 61.90 (10.33) | 409 (58.3%) | 38 (7.4%) | 428 (76.1%) | 468 (92.9%) | 28.01 (5.97) | 15  (8.8%) | 256 (43.0%) | 59 (11.6%) | 326 (63.3%) | 76 (16.6%) |
| Became co-living (n=266) | 61.88 (9.89) | 215 (69.5%) | 8 (2.6%) | 199 (71.9%) | 255 (98.4%) | 28.21 (6.75) | 7  (1.6%) | 134 (43.4%) | 38 (15.1%) | 163 (59.5%) | 44 (17.4%) |
| **Men** | | | | | | | | | | | |
| **Marital Transitions** |  |  |  |  |  |  |  |  |  |  |  |
| Remained partnered (n=6127) | 58.74 (9.69) | 5170 (69.9%) | 1563 (25.2%) | 5763 (91.1%) | 5543 (94.0%) | 28.28 (4.70) | 496 (9.9%) | 2997 (45.6%) | 545 (11.2%) | 3978 (63.5%) | 1204 (21.6%) |
| Remained non-partnered  (n=1185) | 60.53 (10.74) | 946 (62.4%) | 56 (4.0%) | 834 (63.7%) | 1107 (96.7%) | 27.87 (5.21) | 84  (7.5%) | 516 (43.6%) | 151 (13.0%) | 728 (62.1%) | 284 (23.7%) |
| Became divorced (n=109) | 52.19 (6.34) | 90 (74.8%) | 31 (28.9%) | 100 (78.9%) | 100 (92.4%) | 27.02 (3.76) | 8  (13.4%) | 50 (43.7%) | 15 (11.8%) | 64 (71.0%) | 26 (24.5%) |
| Became widowed (n=93) | 66.18 (11.05) | 71 (58.3%) | 12 (10.4%) | 79 (85.2%) | 88 (97.7%) | 28.03 (4.84) | x | 31 (27.0%) | 14 (22.3%) | 69 (75.8%) | 28  (38.1%) |
| Became partnered (n=191) | 56.18 (8.69) | 158 (76.7%) | 30 (12.7%) | 167 (88.1%) | 174 (96.4%) | 26.67 (3.53) | 25 (16.9%) | 97 (40.6%) | 15 (14.2%) | 128 (58.7%) | 48  (27.3%) |
| Uncertain transitions (n=184) | 60.01 (9.32) | 141 (63.0%) | 21 (8.9%) | 139 (73.5%) | 172 (95.1%) | 28.49 (4.29) | 17 (11.9%) | 71 (28.0%) | 11 (7.9%) | 116 (58.1%) | 41 (19.7%) |
| **Cohabitation transitions** | | | | | | | | | | | |
| Remained co-living (n=6548) | 58.52 (9.63) | 5514 (69.6%) | 1632 (24.5%) | 6117 (90.3%) | 5932 (94.1%) | 28.25 (4.70) | 547 (10.2%) | 3210 (45.1%) | 580 (11.0%) | 4236 (63.0%) | 1300 (21.9%) |
| Remained lone-living (n=916) | 62.01 (10.67) | 718 (61.5%) | 38 (2.7%) | 617 (61.3%) | 861 (96.7%) | 28.01 (5.08) | 57  (7.0%) | 370 (37.8%) | 125 (14.8%) | 567 (65.2%) | 212  (19.4%) |
| Became lone-living (n=266) | 59.98 (11.45) | 206 (66.3%) | 29 (13.3%) | 216 (75.6%) | 245 (95.3%) | 26.87 (4.81) | 18  (7.1%) | 114 (49.1%) | 28 (12.6%) | 180 (70.1%) | 73 (28.7%) |
| Became co-living(n=134) | 59.75 (10.15) | 116 (72.4%) | 10 (6.8%) | 109 (70.3%) | 123 (96.9%) | 27.35 (3.86) | 7  (2.3%) | 52 (39.2%) | 13 (15.9%) | 84 (59.5%) | 37 (36.1%) |

Descriptive statistics (frequencies (%) and means (SD)) were calculated using CLSA survey inflation weights. All variables were reported at baseline, except for fruit intake at follow-up 2. 1 The highest education level was post-secondary graduation [university degree]. 2 The highest income was ≥ C$150000. X, value suppressed due to cell size < 5.

**Supplemental Table S4. Sensitivity analysis of the associations between marital transitions and non-daily vegetable intake among aging women and men in the Canadian Longitudinal Study on Aging (2011-21).**

|  | Model A: exclude potato | | Model B: + oral health | | Model C: + physical activity | | Model D: + life satisfaction | | Model E: + reproductive factors | | Model F:  + social ties | |  |
| --- | --- | --- | --- | --- | --- | --- | --- | --- | --- | --- | --- | --- | --- |
| **Marital transitions** | **OR** | **CI95** | **OR** | **CI95** | **OR** | **CI95** | **OR** | **CI95** | **OR** | **CI95** | **OR** | **CI95** |  |
| **Women** | | | | | | | | | | | | | |
| Remained non-partnered | **1.18*** | **[1.04, 1.34]** | **1.21**** | **[1.07, 1.38]** | **1.22**** | **[1.08, 1.39]** | **1.19**** | **[1.04, 1.35]** | **1.18*** | **[1.02, 1.36]** | **1.29**** | **[1.11, 1.52]** |  |
| Became divorced | 1.31 | [0.82, 2.10] | 1.27 | [0.79, 2.05] | 1.35 | [0.84, 2.15] | 1.32 | [0.83, 2.10] | 1.29 | [0.79, 2.10] | 1.39 | [0.88, 2.20] |  |
| Became widowed | 1.22 | [0.84, 1.79] | 1.40 | [0.96, 2.02] | 1.42 | [0.98, 2.07] | 1.39 | [0.96, 2.02] | 1.32 | [0.90, 1.94] | 1.38 | [0.95, 2.00] |  |
| Became partnered | 1.45 | [0.99, 2.13] | 1.45 | [0.98, 2.13] | 1.43 | [0.97, 2.10] | 1.44 | [0.98, 2.11] | 1.44 | [0.98, 2.13] | **1.52*** | **[1.03, 2.24]** |  |
| Uncertain transitions | 1.34 | [0.99, 1.81] | 1.33 | [0.98, 1.81] | **1.37*** | **[1.01, 1.86]** | 1.31 | [0.97, 1.78] | 1.32 | [0.97, 1.80] | **1.41*** | **[1.03, 1.92]** |  |
| **Men** | | | | | | | | | | | | | |
| Remained non-partnered | 1.07 | [0.91, 1.25] | 1.08 | [0.92, 1.27] | 1.07 | [0.91, 1.25] | 1.04 | [0.89, 1.22] | - | - | 1.13 | [0.94, 1.36] |  |
| Became divorced | **1.96**** | **[1.28, 2.99]** | **1.99**** | **[1.29, 3.07]** | **2.00**** | **[1.30, 3.08]** | **1.89**** | **[1.24, 2.90]** | **-** | **-** | **1.92**** | **[1.26, 2.94]** |  |
| Became widowed | **1.73 *** | **[1.05, 2.87]** | **2.03**** | **[1.23, 3.38]** | **2.00**** | **[1.21, 3.29]** | **1.84*** | **[1.12, 3.02]** | **-** | **-** | **1.90*** | **[1.16, 3.11]** |  |
| Became partnered | 1.31 | [0.93, 1.85] | 1.35 | [0.96, 1.92] | 1.32 | [0.94, 1.87] | 1.26 | [0.88, 1.78] | - | - | 1.37 | [0.97, 1.93] |  |
| Uncertain transitions | 1.03 | [0.73, 1.46] | 1.11 | [0.78, 1.56] | 1.10 | [0.78, 1.56] | 1.08 | [0.76, 1.52] | - | - | 1.12 | [0.79, 1.58] |  |

Gender-specific odds ratios (95% CIs) obtained by mixed-effects logistic regression with an interaction term (gender x marital transitions) on the sample with baseline daily vegetable intake. Reference is remained partnered. Model A excludes potato from total vegetable intake (n=15,503). Model B further adjusts for oral health (n=15,520). Model C adjusts for physical activity (n=15,547). Model D adjusts for life satisfaction (n=15,563). Model E includes parity, menopause, and hormone replacement therapy (n=9097). Model F also includes baseline social network, social participation, and living arrangement (n=15,718). *p<0.05; ** p<0.01.

**Supplemental Table S5. Sensitivity analysis of the associations between cohabitation transitions and non-daily vegetable intake among aging women and men in the Canadian Longitudinal Study on Aging (2011-21).**

|  | Model A: exclude potato | | Model B: + oral health | | Model C: + physical activity | | Model D: + life satisfaction | | Model E: + reproductive factors | | Model F:  + social ties | |
| --- | --- | --- | --- | --- | --- | --- | --- | --- | --- | --- | --- | --- |
| **Cohabitation transitions** | **OR** | **CI95** | **OR** | **CI95** | **OR** | **CI95** | **OR** | **CI95** | **OR** | **CI95** | **OR** | **CI95** |
| **Women** | | | | | | | | | | | | |
| Remained lone-living | 1.08 | [0.94, 1.24] | 1.11 | [0.97, 1.27] | 1.11 | [0.97, 1.27] | 1.09 | [0.95, 1.24] | 1.04 | [0.90, 1.21] | 0.94 | [0.79, 1.12] |
| Became lone-living | 1.21 | [0.96, 1.52] | 1.19 | [0.94, 1.51] | 1.23 | [0.98, 1.56] | 1.20 | [0.95, 1.52] | 1.18 | [0.93, 1.50] | 1.15 | [0.91, 1.46] |
| Became co-living | 1.03 | [0.73, 1.45] | 0.99 | [0.70, 1.40] | 0.98 | [0.70, 1.38] | 1.00 | [0.71, 1.40] | 0.98 | [0.69, 1.38] | 0.87 | [0.61, 1.24] |
| **Men** | | | | | | | | | | | | |
| Remained lone-living | 1.03 | [0.87, 1.23] | 1.02 | [0.86, 1.22] | 1.00 | [0.84, 1.20] | 0.99 | [0.83, 1.18] | - | - | 0.83 | [0.67, 1.03] |
| Became lone-living | **1.38*** | **[1.02, 1.86]** | **1.52**** | **[1.13, 2.05]** | **1.49**** | **[1.11, 2.01]** | **1.37*** | **[1.02, 1.84]** | - | - | 1.33 | [0.98, 1.79] |
| Became co-living | 1.47 | [0.98, 2.20] | **1.53*** | **[1.03, 2.29]** | **1.50*** | **[1.00, 2.25]** | 1.46 | [0.97, 2.20] | - | - | 1.31 | [0.87, 1.99] |

Gender-specific odds ratios (95% CIs) obtained by mixed-effects logistic regression with an interaction term (gender x cohabitation transitions) on the sample with baseline daily vegetable intake. Reference is remained co-living. Model A excludes potato from total vegetable intake (n=15,448). Model B further adjusts for oral health (n=15,471). Model C adjusts for physical activity (n=15,498). Model D adjusts for life satisfaction (n=15,508). Model E includes parity, menopause, and hormone replacement therapy (n=9067). Model F also includes baseline social network, social participation, and marital status (n=15,663). *p<0.05; ** p<0.01.

| **A**  **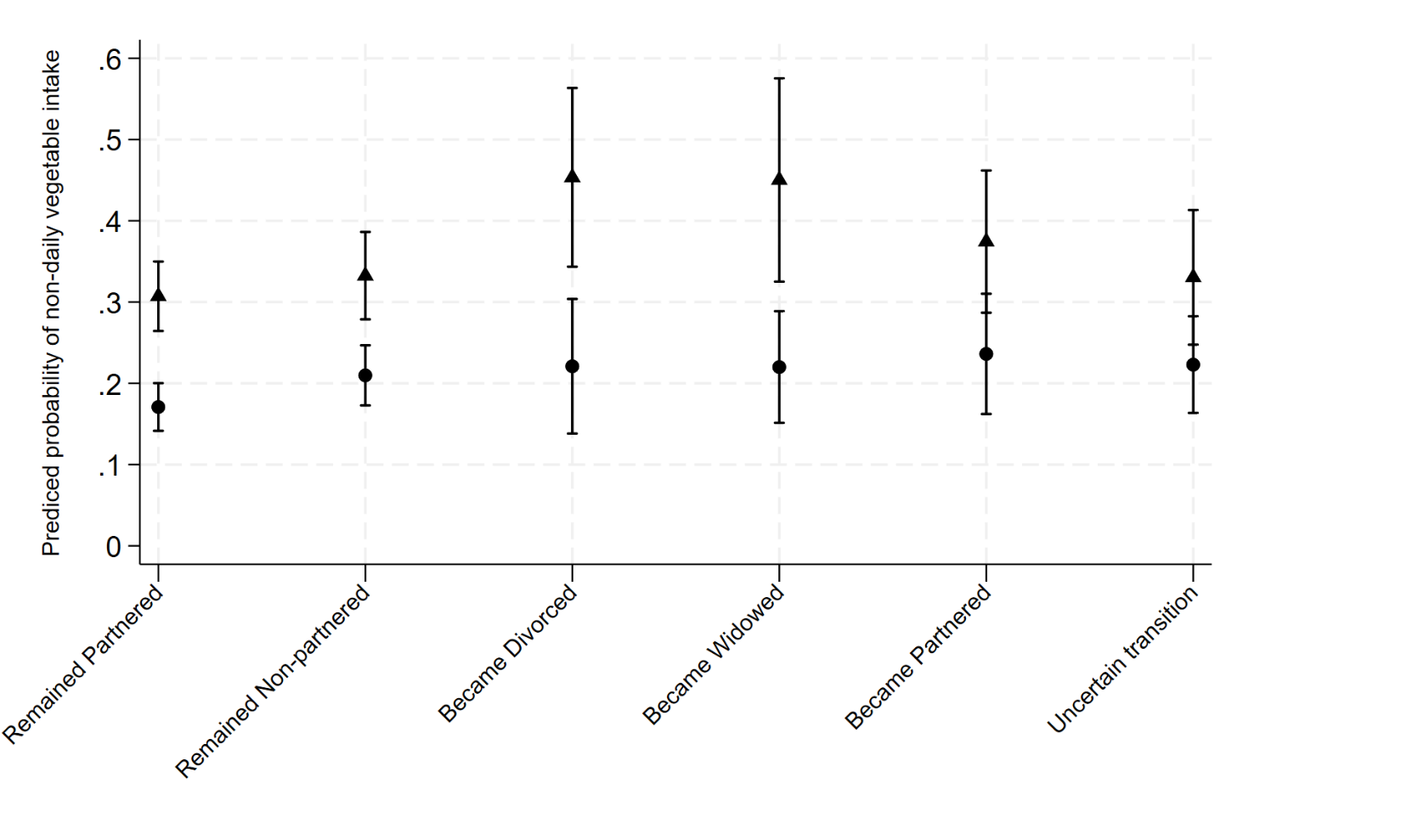** |
| --- |
| **B**  **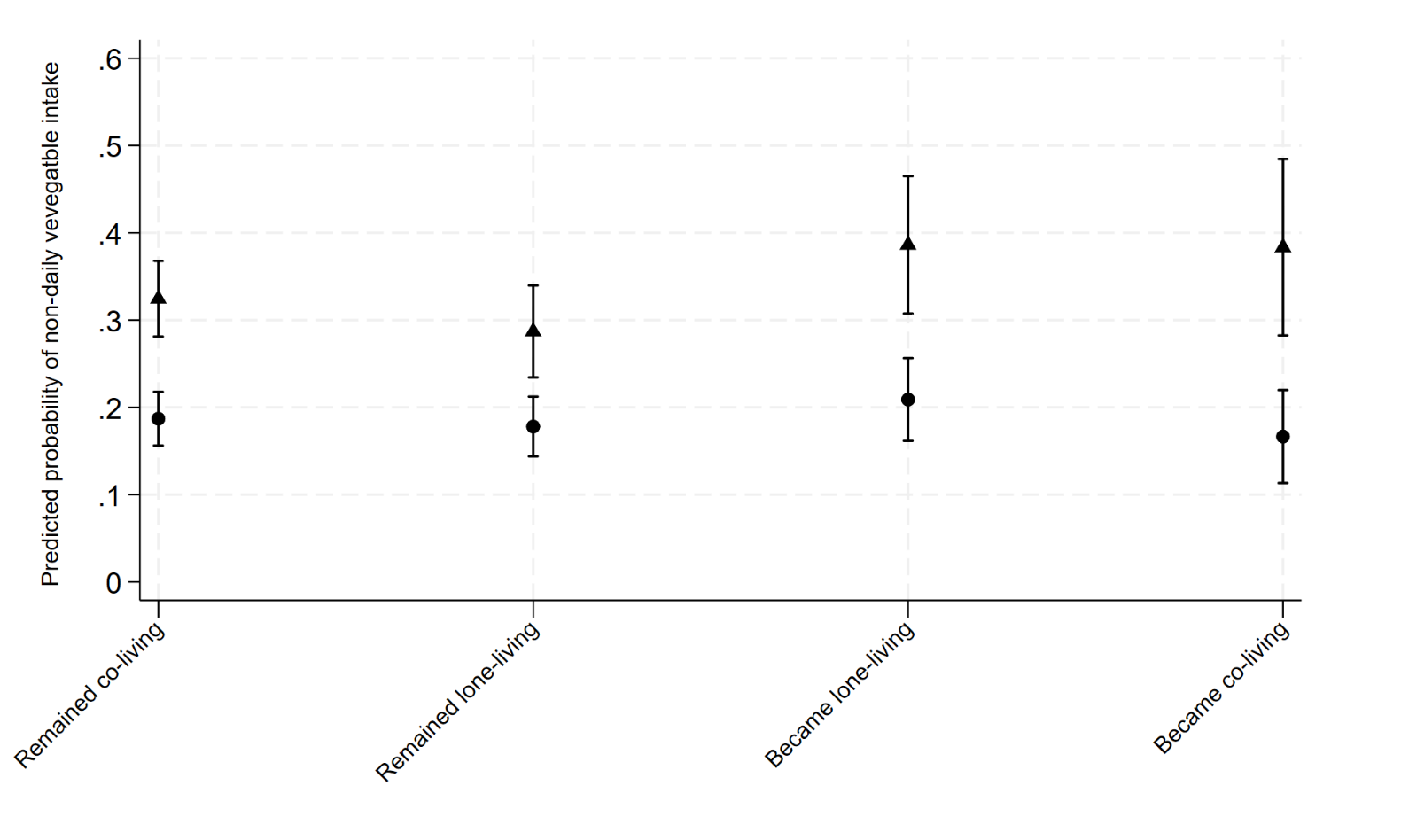** |
| **Supplemental Figure S3. Average predicted probability of non-daily vegetable intake associated with changes in close social ties among aging women and men in the Canadian Longitudinal Study on Aging (2011-21), independent of other social ties.** Triangles represent estimates for men, and circles represent estimates for women, with corresponding 95% confidence intervals. Panel A, marital transitions; Panel B, cohabitation transitions. |

**Supplemental Table S6. Sensitivity analysis of the associations between marital transitions and non-daily fruit intake among aging women and men in the Canadian Longitudinal Study on Aging (2011-21).**

|  | Model A: + oral health | | Model B: + physical activity | | Model C: + life satisfaction | | Model D: + reproductive factors | | Model E: + BL social ties | |
| --- | --- | --- | --- | --- | --- | --- | --- | --- | --- | --- |
| **Marital transitions** | **OR** | **CI95** | **OR** | **CI95** | **OR** | **CI95** | **OR** | **CI95** | **OR** | **CI95** |
| **Women** | | | | | | | | | | |
| Remained non-partnered | 1.03 | [0.90, 1.19] | 1.04 | [0.90, 1.19] | 0.99 | [0.86, 1.14] | 1.05 | [0.90, 1.22] | 1.11 | [0.94, 1.31] |
| Became divorced | **1.65*** | **[1.08, 2.54]** | **1.73*** | **[1.14, 2.64]** | **1.70**** | **[1.13, 2.58]** | **1.84**** | **[1.21, 2.79]** | **1.79**** | **[1.18, 2.72]** |
| Became widowed | 0.85 | [0.54, 1.37] | 0.86 | [0.54, 1.36] | 0.82 | [0.52, 1.31] | 0.90 | [0.56, 1.44] | 0.83 | [0.52, 1.32] |
| Became partnered | 1.10 | [0.73, 1.68] | 1.10 | [0.72, 1.67] | 1.08 | [0.71, 1.64] | 1.09 | [0.72, 1.67] | 1.14 | [0.75, 1.74] |
| Uncertain transitions | 1.34 | [0.97, 1.86] | 1.35 | [0.98, 1.87] | 1.24 | [0.89, 1.72] | 1.22 | [0.87, 1.70] | 1.34 | [0.96, 1.87] |
| **Men** | | | | | | | | | | |
| Remained non-partnered | **1.20*** | **[1.02, 1.41]** | **1.19*** | **[1.02, 1.40]** | **1.17** | **[0.99, 1.37]** | **-** | **-** | **1.28*** | **[1.06, 1.54]** |
| Became divorced | 1.10 | [0.70, 1.75] | 1.10 | [0.70, 1.74] | 1.09 | [0.69, 1.71] | - | - | 1.14 | [0.72, 1.79] |
| Became widowed | **2.10**** | **[1.31, 3.20]** | **2.01**** | **[1.27, 3.17]** | **1.92**** | **[1.21, 3.05]** | **-** | **-** | **2.00**** | **[1.26, 3.16]** |
| Became partnered | 1.29 | [0.92, 1.82] | 1.29 | [0.92, 1.81] | 1.23 | [0.87, 1.73] | - | - | 1.38 | [0.98, 1.94] |
| Uncertain transitions | 1.01 | [0.70, 1.47] | 1.01 | [0.69, 1.47] | 1.06 | [0.74, 1.53] | - | - | 1.13 | [0.78, 1.63] |

Gender-specific odds ratios (95% CIs) obtained by mixed-effects logistic regression with an interaction term (gender x marital transitions) on the sample with BL daily fruit intake. Reference is remained partnered. Model A further adjusts for oral health (n=17861). Model B adjusts for physical activity (n=17885). Model C adjusts for life satisfaction (n=17887). Model D includes parity, menopause, and hormone replacement therapy (n=10066). Model E also includes BL social network, social participation, and living arrangement (n=18066). *p<0.05; ** p<0.01.

**Supplemental Table S7. Sensitivity analysis of the associations between cohabitation transitions and non-daily fruit intake among aging women and men in the Canadian Longitudinal Study on Aging (2011-21).**

|  | Model A: + oral health | | Model B: + physical activity | | Model C: + life satisfaction | | Model D: +  reproductive factors | | Model E: + BL social ties | |
| --- | --- | --- | --- | --- | --- | --- | --- | --- | --- | --- |
| **Cohabitation transitions** | **OR** | **CI95** | **OR** | **CI95** | **OR** | **CI95** | **OR** | **CI95** | **OR** | **CI95** |
| **Women** | | | | | | | | | | |
| Remained lone-living | 0.87 | [0.75, 1.01] | 0.86 | [0.74, 1.00] | **0.84*** | **[0.72, 0.98]** | 0.86 | [0.73, 1.01] | **0.77**** | **[0.64, 0.93]** |
| Became lone-living | 1.02 | [0.79, 1.32] | 1.05 | [0.81, 1.36] | 1.00 | [0.78, 1.30] | 1.01 | [0.77, 1.31] | 0.93 | [0.66, 1.16] |
| Became co-living | 1.14 | [0.81, 1.60] | 1.13 | [0.81, 1.59] | 1.12 | [0.80, 1.57] | 1.14 | [0.81, 1.61] | 1.00 | [0.66, 1.36] |
| **Men** | | | | | | | | | | |
| Remained lone-living | 1.14 | [0.96, 1.36] | 1.13 | [0.95, 1.35] | 1.12 | [0.94, 1.34] | - | - | 1.00 | [0.80, 1.23] |
| Became lone-living | **1.48**** | **[1.11, 1.98]** | **1.44*** | **[1.08, 1.92]** | **1.40**** | **[1.05, 1.86]** | **-** | **-** | **1.37*** | **[1.03, 1.83]** |
| Became co-living | **1.53*** | **[1.04, 2.27]** | **1.52*** | **[1.03, 2.24]** | 1.37 | [0.92, 2.04] | - | - | 1.34 | [0.89, 2.02] |

Gender-specific odds ratios (95% CIs) obtained by mixed-effects logistic regression with an interaction term (gender x cohabitation transitions) on the sample with BL daily fruit intake. Reference is remained co-living. Model A further adjusts for oral health (n=17805). Model B adjusts for physical activity (n=17829). Model C adjusts for life satisfaction (n=17825). Model D includes parity, menopause, hormone replacement therapy (n=10030). Model E also includes BL social network, social participation, and marital status (18005). *p<0.05; ** p<0.01.

| \| **A**  **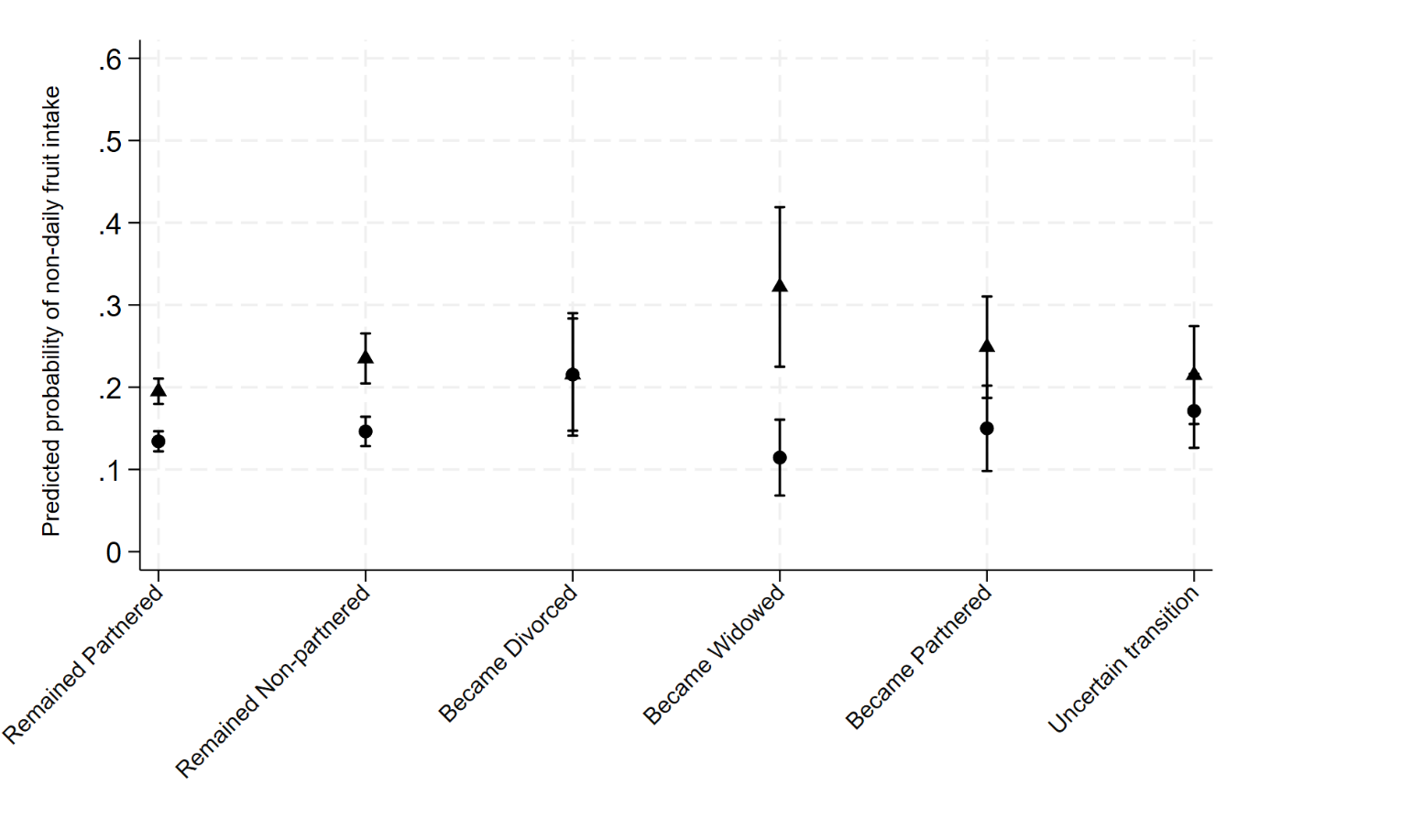**  **B**  **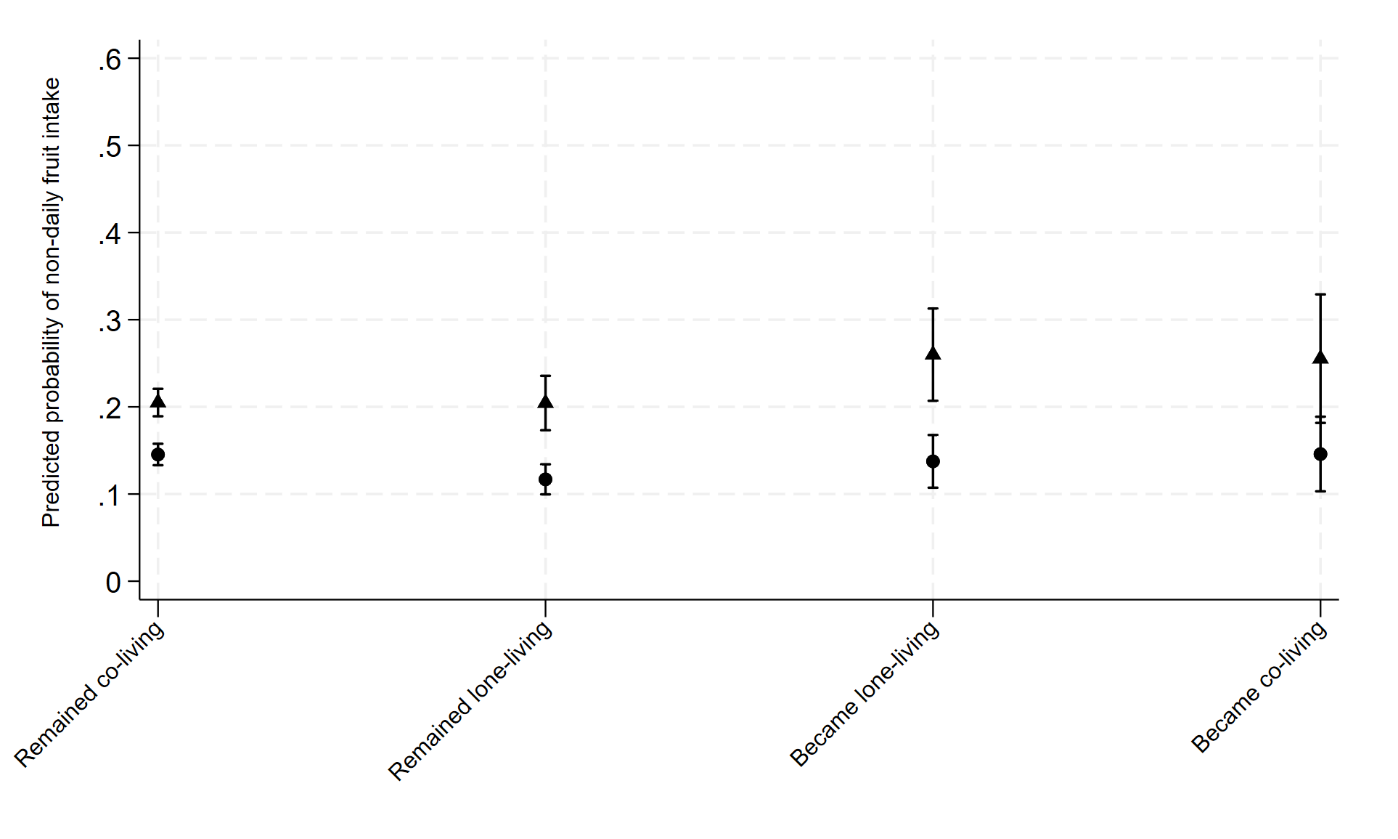** \| \| --- \| |
| --- | --- |

**Supplemental Figure S4. Average predicted probability of non-daily fruit intake for sensitivity analysis of changes in close social ties while adjusting for other social ties among aging women and men in the Canadian Longitudinal Study on Aging (2011-21).** Triangles represent estimates for men, and circles represent estimates for women, with corresponding 95% confidence intervals. Panel A, marital transitions; Panel B, cohabitation transitions.

**Supplemental References**

1. Raina PS, Wolfson C, Kirkland SA, Griffith LE, Oremus M, Patterson C, et al. The Canadian longitudinal study on aging (CLSA). Can J Aging. 2009;28(3):221-9.

2. Raina P, Wolfson C, Kirkland S. Canadian longitudinal study on aging (CLSA) protocol version 3.0, 2008. Accessed April 26, 2024. <https://www.clsa-elcv.ca/researchers>.

3. 2023 CLSA Sampling and Computation of Response Rates and Sample Weights for the Tracking (Telephone Interview) Participants and Comprehensive Participants (Version 2.0). Accessed April 26, 2024. <https://www.clsa-elcv.ca/doc/5130>.

4. Shatenstein B, Payette H. Evaluation of the Relative Validity of the Short Diet Questionnaire for Assessing Usual Consumption Frequencies of Selected Nutrients and Foods. Nutrients. 2015;7(8):6362-74.

5. Canada's food guide. Eat vegetables and fruits. Accessed March 20, 2024. <https://food-guide.canada.ca/en/healthy-eating-recommendations/make-it-a-habit-to-eat-vegetables-fruit-whole-grains-and-protein-foods/eat-vegetables-and-fruits/>.

6. NIH National Cancer Institute. (2021). Identifying Extreme Exposure Values. Accessed March 20, 2024. <https://epi.grants.cancer.gov/nhanes/dietscreen/scoring/current/identify.html>.

7. Wilson SE. Marriage, gender and obesity in later life. Economics & Human Biology. 2012;10(4):431-53.

8. Ceolin G, Veenstra G, Khan NA, Madani Civi R, Mehranfar S, Conklin AI. Adverse changes in close social ties in aging women and men: A population-based longitudinal study of the CLSA (2011–2021). Archives of Gerontology and Geriatrics. 2025;130:105720.

9. Textor J, van der Zander B, Gilthorpe MS, Liskiewicz M, Ellison GT. Robust causal inference using directed acyclic graphs: the R package 'dagitty'. Int J Epidemiol. 2016;45(6):1887-94.

10. Ding D, Gale J, Bauman A, Phongsavan P, Nguyen B. Effects of divorce and widowhood on subsequent health behaviours and outcomes in a sample of middle-aged and older Australian adults. Sci Rep. 2021;11(1):15237.

11. Eng PM, Kawachi I, Fitzmaurice G, Rimm EB. Effects of marital transitions on changes in dietary and other health behaviours in US male health professionals. J Epidemiol Community Health. 2005;59(1):56-62.

12. Lee S, Cho E, Grodstein F, Kawachi I, Hu FB, Colditz GA. Effects of marital transitions on changes in dietary and other health behaviours in US women. Int J Epidemiol. 2005;34(1):69-78.

13. Noguchi T, Kondo F, Nishiyama T, Otani T, Nakagawa-Senda H, Watanabe M, et al. The Impact of Marital Transitions on Vegetable Intake in Middle-aged and Older Japanese Adults: A 5-year Longitudinal Study. J Epidemiol. 2022;32(2):89-95.

14. Plessz M, Gueguen A. Who Benefits from Living in a Couple?

A Longitudinal Study of Eating Practices at the Intersection of Gender, Conjugal Situation, and Social Status. Revue française de sociologie (English Edition). 2017;58(4):1-29.

15. Vinther JL, Conklin AI, Wareham NJ, Monsivais P. Marital transitions and associated changes in fruit and vegetable intake: Findings from the population-based prospective EPIC-Norfolk cohort, UK. Soc Sci Med. 2016;157:120-6.

16. Wilcox S, Evenson KR, Aragaki A, Wassertheil-Smoller S, Mouton CP, Loevinger BL. The effects of widowhood on physical and mental health, health behaviors, and health outcomes: The Women's Health Initiative. Health Psychol. 2003;22(5):513-22.

17. Ikram MA. The disjunctive cause criterion by VanderWeele: An easy solution to a complex problem? Eur J Epidemiol. 2019;34(3):223-4.

18. Pollack CE, Chideya S, Cubbin C, Williams B, Dekker M, Braveman P. Should health studies measure wealth? A systematic review. Am J Prev Med. 2007;33(3):250-64.

19. Bassim C, Mayhew AJ, Ma J, Kanters D, Verschoor CP, Griffith LE, et al. Oral Health, Diet, and Frailty at Baseline of the Canadian Longitudinal Study on Aging. J Am Geriatr Soc. 2020;68(5):959-66.
